# Aromatic Rings Commonly Used in Medicinal Chemistry: Force Fields Comparison and Interactions With Water Toward the Design of New Chemical Entities

**DOI:** 10.3389/fphar.2018.00395

**Published:** 2018-04-24

**Authors:** Marcelo D. Polêto, Victor H. Rusu, Bruno I. Grisci, Marcio Dorn, Roberto D. Lins, Hugo Verli

**Affiliations:** ^1^Grupo de Bioinformática Estrutural, Centro de Biotecnologia, Universidade Federal do Rio Grande do Sul, Porto Alegre, Brazil; ^2^Swiss National Supercomputing Centre, Lugano, Switzerland; ^3^Instituto de Informática, Universidade Federal do Rio Grande do Sul, Porto Alegre, Brazil; ^4^Instituto Aggeu Magalhães, Fundação Oswaldo Cruz, Recife, Brazil

**Keywords:** drug design, GROMOS, aromatic rings, functional groups, interactions

## Abstract

The identification of lead compounds usually includes a step of chemical diversity generation. Its rationale may be supported by both qualitative (SAR) and quantitative (QSAR) approaches, offering models of the putative ligand-receptor interactions. In both scenarios, our understanding of which interactions functional groups can perform is mostly based on their chemical nature (such as electronegativity, volume, melting point, lipophilicity etc.) instead of their dynamics in aqueous, biological solutions (solvent accessibility, lifetime of hydrogen bonds, solvent structure etc.). As a consequence, it is challenging to predict from 2D structures which functional groups will be able to perform interactions with the target receptor, at which intensity and relative abundance in the biological environment, all of which will contribute to ligand potency and intrinsic activity. With this in mind, the aim of this work is to assess properties of aromatic rings, commonly used for drug design, in aqueous solution through molecular dynamics simulations in order to characterize their chemical features and infer their impact in complexation dynamics. For this, common aromatic and heteroaromatic rings were selected and received new atomic charge set based on the direction and module of the dipole moment from MP2/6-31G^*^ calculations, while other topological terms were taken from GROMOS53A6 force field. Afterwards, liquid physicochemical properties were simulated for a calibration set composed by nearly 40 molecules and compared to their respective experimental data, in order to validate each topology. Based on the reliance of the employed strategy, we expanded the dataset to more than 100 aromatic rings. Properties in aqueous solution such as solvent accessible surface area, H-bonds availability, H-bonds residence time, and water structure around heteroatoms were calculated for each ring, creating a database of potential interactions, shedding light on features of drugs in biological solutions, on the structural basis for bioisosterism and on the enthalpic/entropic costs for ligand-receptor complexation dynamics.

## 1. Introduction

The development of a drug is a multi step process, usually starting with the identification of hit compounds. The challenging task of optimizing these compounds into leads and finally into drugs is commonly facilitated by computer aided drug design (CADD) techniques (Anderson, 2003; Sliwoski et al., 2013; Bajorath, 2015). With the growing information on protein structure on the last years, structure based drug design (SBDD) has become a significant tool for hit discovery (Anderson, 2003; Lounnas et al., 2013; Lionta et al., 2014). When structural information of the receptor is absent, molecular fingerprints of approved drugs are also used to search for new ligands in a process also known as ligand based drug design (LBDD) (Lee et al., 2011). Nevertheless, there are still considerable challenges associated to the predictiveness of ligand potency and affinity via computational methods (Paul et al., 2010; Csermely et al., 2012).

In general, optimization of lead compounds is based in qualitative or quantitative structure-activity relationships (SAR or QSAR, respectively) (Shahlaei, 2013). These relationships are usually based in molecular descriptors to predict ligand pharmacodynamics and pharmacokinetics, such as log*P* to access lipophilicity, log*S* to access solubility or p*K*a to access the ionic state of a compound, along with other topological, geometrical and physicochemical descriptors (Danishuddin and Khan, 2016). While some correlations have reasonable power of predictiveness, many descriptors have no biological meaning and can mislead the optimization process. As highlighted by Hopkins et al. (2014), high-throughput screening methods have been linked to the rise of hits with inflated physicochemical properties during the optimization process (Keserü and Makara, 2009). Also, recent reviews have shown an increase of molar mass in the recent medicinal chemistry efforts (Leeson and Springthorpe, 2007) and many authors correlate this strategy with the likelihood of poor results of such compounds (Gleeson, 2008; Waring, 2009, 2010; Gleeson et al., 2011).

Many chemical moieties are regularly used in medicinal chemistry to produce chemical diversity (Bemis and Murcko, 1996; Welsch et al., 2010; Taylor et al., 2014), a practice well-known as fragment based drug design (FBDD), and its use for pharmacophore modeling and to prevent high toxicity is not recent (Gao et al., 2010). Particularly, aromatic rings are extensively used in drugs due to their well known synthetic and modification paths (Aldeghi et al., 2014). For example, at least, one aromatic ring can be found in 99% of a database containing more than 3,500 evaluated by the medicinal chemistry department of Pfizer, AstraZeneca (AZ) and GlaxoSmithKlin (GSK) (Roughley and Jordan, 2011). Still, little is known about their chemical features in biological solution, such as H-bonds availability, lifetime of H-bonds, solvent accessibility, and conformational ensemble. In this sense, molecular dynamics (MD) simulations can provide useful information with atomistic resolution and access the aforementioned features of chemical groups in water, providing fundamental data to drive medicinal chemistry approaches.

Still, dynamical properties of chemical moieties in biological solution are usually neglected in drug design and very difficult to access (Ferenczy and Keseru, 2010; Reynolds and Holloway, 2011; Hopkins et al., 2014). Even though MD simulations have been used in medicinal chemistry to generate different receptor conformers and to validate binding poses predicted by docking (Zhao and Caflisch, 2015; Ganesan et al., 2017), simulations of free ligand in solution is rarely used to access the conformational ensemble and energies associated with solvation due to the challenge on solving conformational flexibility and internal energies (Butler et al., 2009; Blundell et al., 2016). When solvated, the enthalpic and entropic costs of disrupting a H-bond or dismantling the entire solvation shell of a ligand can be the determinant step to provide the proper energy of binding (Biela et al., 2012; Blundell et al., 2013; Mondal et al., 2014). Yet, free-energy of binding is often predicted via geometrical or alchemical transformations (Zwanzig, 1954; Aqvist et al., 1994; Woo and Roux, 2005; Gumbart et al., 2013), alongside with recent developments in funnel metadynamics (Limongelli et al., 2013). More recently, thermodynamical features of ligands have been experimentally investigated in order to enhance binding and efficiency (Freire, 2009; Ferenczy and Keseru, 2010; Reynolds and Holloway, 2011). Ligand features such as H-bonds lifetime, effects of vicinity in H-bonds availability and strength, accessible surface area and water structure around binding sites can provide substantial information for designing new molecular entities (Blundell et al., 2016).

Different force fields have been used for drug design purposes, such as MMF94 (Halgren, 1996), OPLS-AA (Jorgensen et al., 1996), and GAFF (Wang et al., 2004). While these force fields parameterized their electrostatic terms using *ab initio* calculations, the GROMOS force fields (derived from the Groningen Molecular Simulation package) used free-energy of solvation as target (Daura et al., 1998; Oostenbrink et al., 2004) to empirically assign atomic partial charges. Thus, in this work, we have chosen the GROMOS force field to simulate the dynamical behavior of 103 aromatic rings (including a calibration subset of 42 molecules) mostly commonly used in drug design and their interactions with solvent in order to access thermodynamical properties in solution. These interactions, in turn, offer a reference for future rational drug design studies, as describe in details how several functional groups interact with their surroundings.

## 2. Methods

### 2.1. Selection of rings

A series of 103 aromatic rings commonly used in drug design were selected for this study (Broughton and Watson, 2004; Jordan and Roughley, 2009; Welsch et al., 2010; Taylor et al., 2014, 2017). Among them, a calibration set of 42 molecules (Table 1), for which physical-chemical properties are known, were selected from the benchmark developed by Caleman et al. (2012). Briefly, both works of Taylor et al. (2014, 2017) employed a detailed search of substructure frequencies from FDA Orange Book and cross referenced with ChEMBL, DrugBank, Nature, Drug Reviews, the FDA Web site, and the Annual Reports in Medicinal Chemistry; the work of Broughton and Watson (2004) employed search of substructure frequencies in MDL Drug Data Report database by using a “Phase II” keyword; and the work of Welsch et al. (2010) have pinpointed privileged scaffolds from natural-products works throughout literature.

**Table 1:**
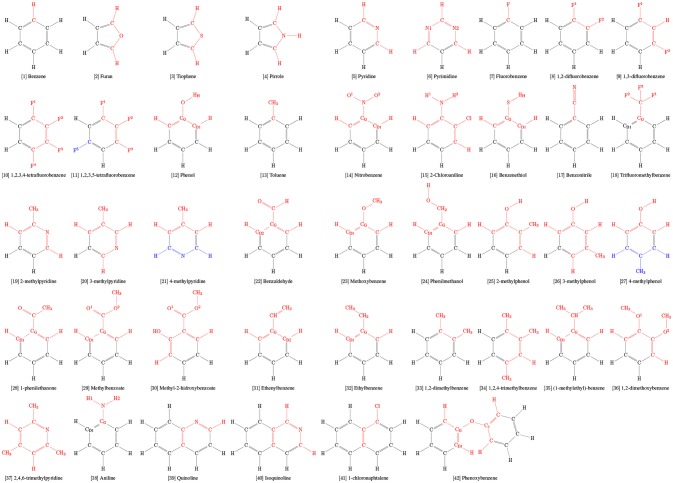
Charge groups (colored) and aromatic rings used as calibration set in this work.

### 2.2. Topology construction

Structures for these aromatic rings were built using Avogadro (Hanwell et al., 2012). Molecular mechanical (MM) topological parameters as bonds, angles, and Lennard-Jones parameters were taken from GROMOS53A6 (Oostenbrink et al., 2004). Due to the well–known good performance of MP2 methods for small aromatic rings (Li et al., 2015; Matczak and Wojtulewski, 2015), atomic partial charges were based on quantum mechanical (QM) calculations using MP2 theory (Møller and Plesset, 1934), 6-31G^*^ (Petersson et al., 1988) basis set and implicit solvent *Polarizable Continuum Model* (PCM) (Mennucci and Tomasi, 1997) followed by a RESP fitting (Bayly et al., 1993). The so obtained partial charges were adjusted in the MM to reproduce the QM dipole moment of the ring. The angle θ formed between the QM and MM model dipole moment vectors was monitored through an in house script to make sure the angle had the lowest value possible, guaranteeing the conservation of the QM dipole moment direction. For our calibration set, the module of the MM partial charges were adjusted to better reproduce the physicochemical properties of the organic liquids. Following the philosophy of charge group assignment, groups were limited, at maximum, to the atoms at the *ortho* position on each ring. In more complex substitution patterns, a superimposition of two charge groups was required to correctly describe the chemical group. In such cases, the Coulombic terms of the overlapping atoms were adjusted to correctly describe the direction of the total dipole moment of the ring. For molecules containing linear constraints (benzonitrile), *virtual sites* were added in order to preserve the total moment of inertia and mass, thus preserving the linearity of these groups (Feenstra et al., 1999).

### 2.3. New torsional potentials

The quantum mechanical torsional profile of every dihedral angle was calculated using Gaussian (Frisch et al., 2016) (RRID:SCR_014897). Molecular structures were built using Avogadro (Hanwell et al., 2012) and their geometry were optimized using Hartree-Fock method (Fock, 1930; Hartree and Hartree, 1935) and basis set 3-21G^*^ (Dobbs and Hehre, 1986). Afterwards, the *Scan* routine was used to calculate the total energy of the molecule conformation for each dihedral orientation, adopting a *tight* convergence criteria, with geometric optimization, MP2/6-31G^*^ and steps of 30°. In order to calculate the torsional profile for molecular mechanics model, dihedral orientations were kept fixed during minimization using restraint forces for the same angles evaluated by quantum calculations. Both profiles were submitted to the Rotational Profiler server (Rusu et al., 2014) to obtain appropriate sets of classical mechanics parameters that provided a better fitting to the QM-obtained torsional profile.

### 2.4. General simulation settings

All simulations were carried out using the GROMACS 5.0.7 package (Abraham et al., 2015) (RRID:SCR_014565). In order to create parameters compatible with the GROMOS family, we have followed previous literature (Daura et al., 1998; Schuler et al., 2001; Oostenbrink et al., 2004) settings: twin-range scheme was used with short- and long-range cutoff distances of 0.8 and 1.4 nm, respectively. Also, the reaction-field method was applied to correct the effects of electrostatic interactions beyond the long-range cutoff distance (Barker and Watts, 1973; Tironi et al., 1995), using the dielectric constant as ε_*RF*_ for organic liquid simulations and ε_*RF*_ = 62 for simulations in water (Heinz et al., 2001; Oostenbrink et al., 2004). The LINCS algorithm (Hess et al., 1997; Hess, 2008) was used to constrain all covalent bonds, using a cubic interpolation, a Fourier grid of 0.12 nm and timestep of 2 fs. Configurations were saved at every 2 ps for analysis.

#### 2.4.1. Organic liquids simulations

In order to build the organic liquid systems, cubic boxes of 2 × 2 × 2 nm were created, each with a single organic molecule. A total of 125 of these boxes were stacked, forming an unique box with conventional periodic boundary conditions treatment of 10 × 10 × 10 nm which was simulated under high pressure (100 bar) to induce liquid phase. The systems were then simulated and equilibrated at 1 bar. Afterwards, the boxes were staggered to obtain systems with 1000 molecules in liquid phase and simulated at 1 bar until the total energy drift converged to values below 0.5 J/(mol × ns × Degrees of Freedom). Such criterion is necessary to make sure that the fluctuating properties could be accurately calculated (Caleman et al., 2012). All simulations were carried out with Berendsen pressure and temperature coupling algorithm due to their efficiency in molecular relaxations (Berendsen et al., 1984), using τ_*T*_ = 0.2 ps and τ_*P*_ = 0.5 ps. When available, experimental values of isothermal compressibility and dielectric constant were used as an additional parameter for liquid simulations. Otherwise, the compressibility of the most chemically similar molecule was used. The experimental dielectric constants from each liquid were also used as parameters in the simulations (Oostenbrink et al., 2004).

In order to calculate the densities of liquids (ρ), simulations at constant pressure were carried out for 10 ns and ρ were calculated using block averages of 5 blocks. Enthalpy of vaporization (Δ*H*_*vap*_) were calculated by block averaging the same 10 ns of liquid simulation to obtain *E*_*pot*_(*l*) and another 100 ns of gas phase simulation using a stochastic dynamics integrator (SD) (Van Gunsteren and Berendsen, 1988) with a single molecule in vacuum, to obtain *E*_*pot*_(*g*) as the equation:

(1) $$\begin{array}{l} \Delta H_{vap}=\left(E_{pot}\left(g\right)+k_{B}T\right)-E_{pot}\left(l\right) \end{array}$$

Aiming to calculate the dielectric constant (ε), the simulation of the liquid boxes from which ρ were obtained were extended up to 60 ns. Convergence calculations of ε were done using running averages and ε were evaluated only after convergence. In order to calculate thermal expansion coefficients (α_*P*_) and classic isobaric heat capacities (*C*_*P*_cla__), three constant pressure simulations were carried out for 5 ns each, with temperatures T, T+10K, and T-10K, for each liquid. The calculations of α_*P*_ and *C*_*P*_cla__ were done using the finite difference method (Kunz and van Gunsteren, 2009):

(2) $$\begin{array}{l} \alpha_{P}\approx \frac{1}{V}{\left(\frac{\partial V}{\partial T}\right)}_{P}\approx -\frac{\ln{\langle \rho \rangle }_{T_{2}}-\ln{\langle \rho \rangle }_{T_{1}}}{T_{2}-T_{1}} \end{array}$$

and:

(3) $$\begin{array}{l} C_{P}\approx {\left(\frac{\partial U}{\partial T}\right)}_{P}\approx \frac{{\langle U\rangle }_{T_{2}}-{\langle U\rangle }_{T_{1}}}{T_{2}-T_{1}} \end{array}$$

In order to calculate isothermal compressibilities (κ_*T*_), three constant volume simulations were carried out for 5 ns each, with pressures 1, 0.9, and 1.1 bar. The calculations of κ_*T*_ was also done using the finite difference method:

(4) $$\begin{array}{l} \kappa_{T}\approx \frac{1}{V}{\left(\frac{\partial V}{\partial P}\right)}_{T}\approx -\frac{\ln\;\rho_{2}-\ln\;\rho_{1}}{{\langle P\rangle }_{\rho_{2}}-{\langle P\rangle }_{\rho_{1}}} \end{array}$$

#### 2.4.2. Solvation free energy simulations

Simulations in water were carried out to evaluate the solvation free energies (Δ*G*_hyd_) of 30 molecules at 1 bar and 298 K. Each aromatic ring (solute) was centered into a cubic box with appropriate dimensions to reproduce the density of SPC water models (0.997 g/cm^3^). In free-energy calculations using thermodynamic integration (TI) method, a coupling parameter λ is used to perturb solute-solvent interactions.

(5) $$\begin{array}{l} \Delta G_{\text{sim}}=\int_{0}^{1}{\langle \frac{\partial H}{\partial \lambda }\rangle }_{\lambda }d\lambda \end{array}$$

in which *H* is the Halmiltonian, λ = 0 refers to the state in which the solute fully interacts with the solvent and λ = 1 refers to the state in which the solute-solvent interactions do not exist. In our setup, Coulombic interactions were decoupled first, and the Lennard-Jones interactions after, using a soft-core potential to avoid issues related to strong Lennard-Jones interactions (Beutler et al., 1994). A soft-core power was set to 1 and α_LJ_ set to 0.5, following recommendations of Shirts and Pande (2005). Both interactions were decoupled using λ values: 0, 0.02, 0.04, 0.07, 0.1, 0.15, 0.2, …, 0.8, 0.85, 0.9, 0.93, 0.96, 0.98, 1, totalizing 50 λ simulations.

Our simulation protocol consisted of an initial steepest-descent minimization, followed by a L-BFGS minimization until a maximum force of 10 kJ/(mol^−1^ nm^−1^) was reached. After, initial velocities were assigned and the systems were equilibrated for 100 ps using a NVT ensemble at each λ. The systems were subjected to another 100 ps of equilibration on a NPT ensemble, using the Parrinello-Rahman pressure coupling algorithm (Parrinello and Rahman, 1981), a τ_*t*_ = 5 ps time constant for coupling and a compressibility of 4.5 × 10^5^ bar^−1^. Finally, production simulations were done using the Langevin integrator (Van Gunsteren and Berendsen, 1988) to sample the 〈∂*H*/∂λ〉_λ_ until convergence. Therefore, simulations time varied between 1 and 5 ns. In addition, the last frame of the production phase of each λ was used as input for the next subsequent λ.

#### 2.4.3. Simulation of rings in water

After an extensive comparison of simulated and experimental physicochemical properties of our calibration set and consequent validation, the same strategy of topological construction was applied to other 61 rings commonly used in drug design (Table 2) for which experimental properties are not available, totalizing 103 aromatic rings in this study. Hence, in order to evaluate chemical features and interactions of aromatic rings with their surroundings, a total set of 103 aromatic was simulated in water, including all 42 molecules present in the calibration set (Table 1). Each solute was placed in a cubic box with a distance of 1.0 nm to its edges. The boxes were then filled with SPC water model and minimized long enough eliminate any possible clashes until convergence at a maximum force of 0.1 kJ/mol × nm. After, the system was equilibrated in a NVT ensemble at 298.15 K using the Nosé-Hoover algorithm (Nosé, 1984) for temperature coupling. Production runs of 250 ns were carried out with temperature and pressure coupling handled by V-rescale (Bussi et al., 2007) and Parrinelo-Rahman (Parrinello and Rahman, 1981) algorithms, using τ_*T*_ = 0.1 ps and τ_*P*_ = 2.0 ps. The GROMACS tools *hbond, rdf*, and *sorient* were used to calculate H-bonds related properties and solvation structure around the heteroatom using a block-averaging approach over 5 box of 50 ns.

**Table 2:**
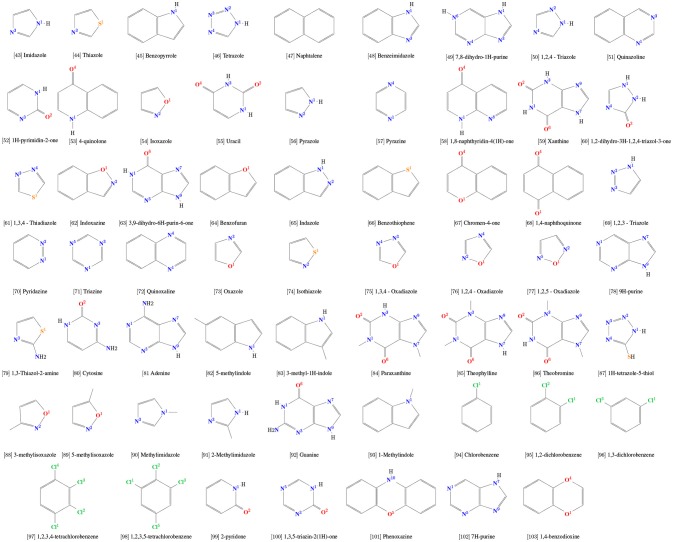
Dataset of aromatic rings evaluated in this work. Heteroatoms are highlighted in colors.

## 3. Results

### 3.1. New torsional profiles

**Figure 1:**
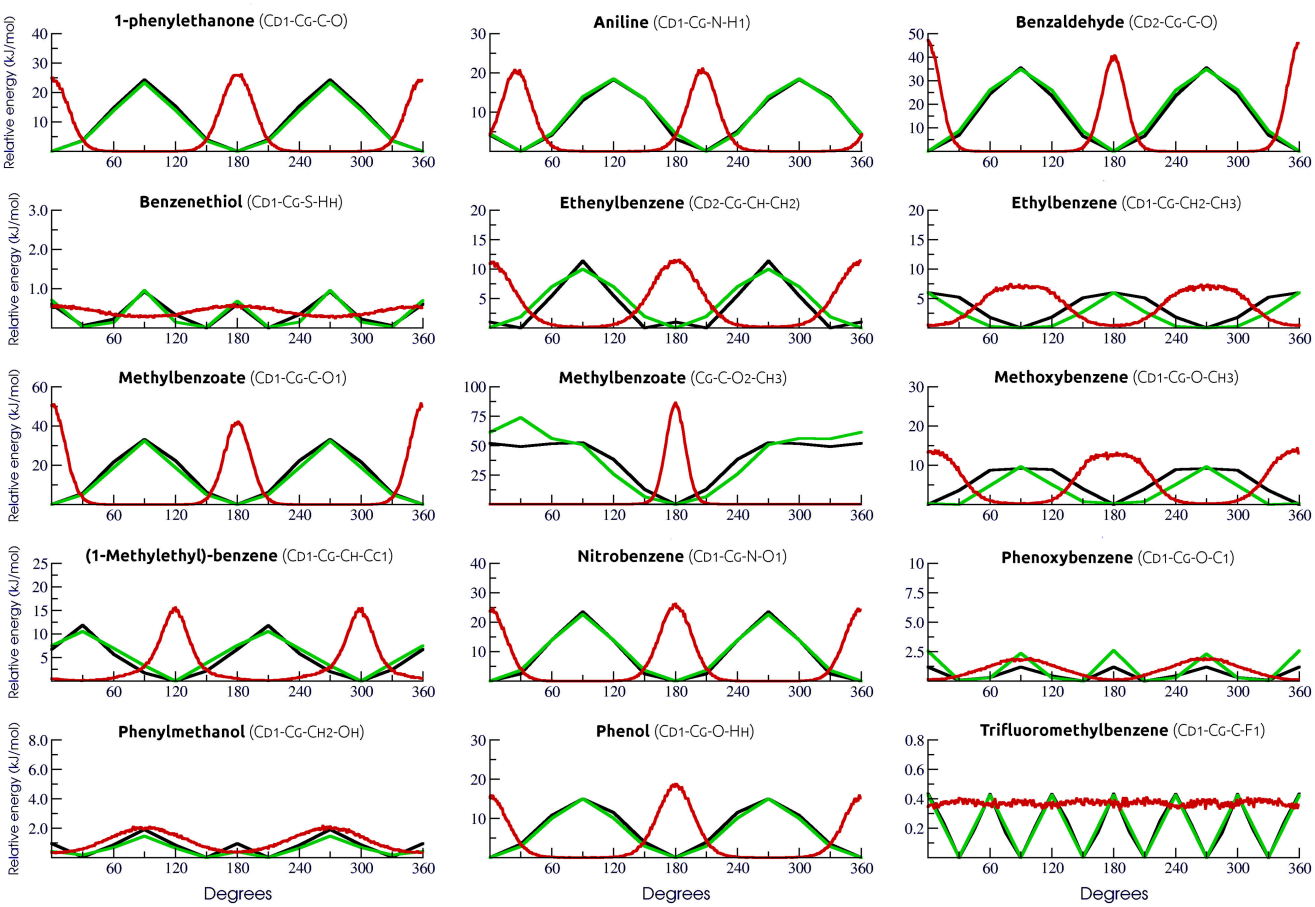
Evaluation of torsional parameters and dihedral distribution. QM and adjusted MM torsional profiles are shown in black and green, respectively. In red, the dihedral distribution during simulations.

In order to accurately describe the torsional angles of the selected aromatic rings, a total of 15 new dihedral potentials were derived by fitting the MM profiles to the corresponding QM-calculated ones (Table S1). Fittings were conducted using the Rotational Profiler server (Rusu et al., 2014). For all cases, the use of new parameters yield almost identical values of minimum and barrier amplitudes to those calculated by QM (Figure 1). Dihedral distribution throughout simulations was also evaluated.

### 3.2. Physical-chemical properties

In order to validate our strategy of topology building, boxes of organic liquids were simulated to obtain physical-chemical properties for each compound. Reference experimental values (Table S2) were used to calculate the absolute error of each property and to guide adjustments on the coulombic terms in order to mitigate deviations. We have calculated the θ angle between QM and MM dipole moments and the final version of our calibration set (Table 1) yielded an average θ angle of 2.5° ± 6.1°, suggesting that our MM models conserve the direction of the QM dipole moment, preserving the electrostatic potential of each molecule.

**Figure 2:**
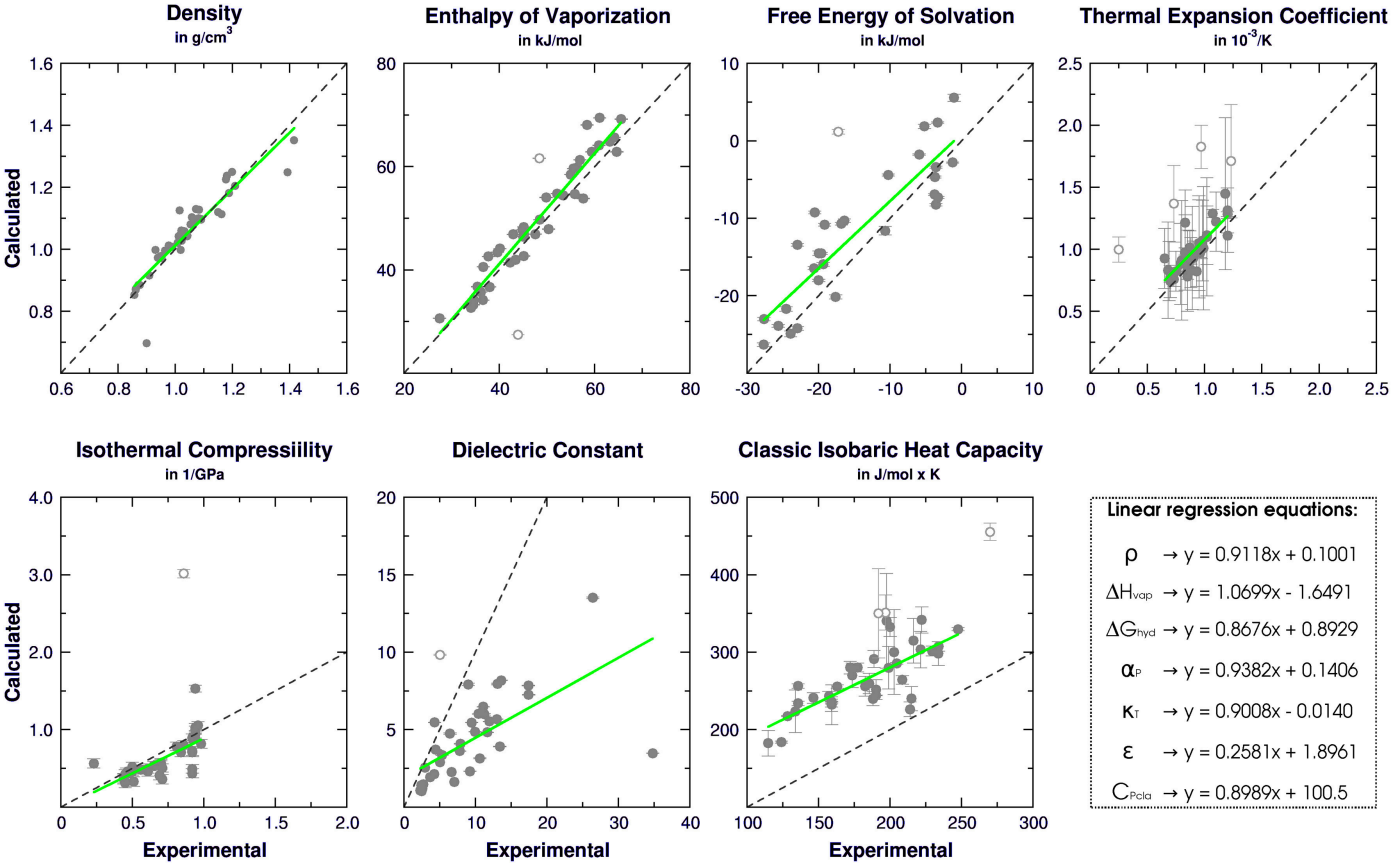
Correlation between experimental and calculated physical-chemical properties of organic liquids for 42 aromatic compounds on the calibration set. Standard deviations are shown as bars, linear regressions are shown as green and empty dots represent outliers.

Following the GROMOS philosophy (Oostenbrink et al., 2004; Horta et al., 2016), density (ρ), enthalpy of vaporization (Δ*H*_vap_), and free energy of solvation (Δ*G*_hyd_) were used as targets for the parametrization, while isothermal coefficient (α_*P*_), isothermal compressibility (κ_*T*_), dielectric constant (ε), and classic isobaric heat capacity (*C*_*P*_cla__) were calculated as benchmarks for GROMOS performance and compared with the results obtained in Caleman et al. (2012) and Horta et al. (2016) (Table 3). Linear regression between experimental and simulated values were calculated in order to access the prediction power of the employed strategy (Figure 2). The equations further reported were calculated excluding outliers (values higher than 2 standard deviations).

**Table 3:** Average deviation between experimental and simulated physicochemical properties of aromatic rings evaluated in our calibration set. Simulated GAFF and OPLS-AA values were obtained from Caleman et al. (2012) and 2016H66 values from Horta et al. (2016). Density (ρ) in g/cm^3^, enthalpy of vaporization (Δ*H*_*vap*_) in kJ/mol, thermal expansion coefficient (α_*P*_) in 10^−3^/K, isothermal compressibility (κ_*T*_) in 1/GPa, dielectric constant (ε), classic isobaric heat capacity (*Cp*_*cla*_) in J/mol × K, and free-energy of solvation (Δ*G*_*hyd*_) in kJ/mol.

**Properties**	**Force field**	**Statistical N**	**Average Dev**.	**St. Dev**.	**R coefficient**
ρ	This work	42	0.008	0.051	0.92
	2016H66	6	0.016	0.019	0.99
	GAFF	40	−0.008	0.045	0.93
	OPLS-AA	40	0.001	0.025	0.98
Δ*H*_*vap*_	This work	42	1.514	4.457	0.96
	2016H66	6	2.257	6.758	0.96
	GAFF	40	2.298	5.419	0.88
	OPLS-AA	40	3.243	5.216	0.90
*Cp*_*cla*_	This work	42	88.201	33.440	0.77
	2016H66	6	98.712	35.232	0.63
	GAFF	37	133.884	40.225	0.84
	OPLS-AA	37	129.397	35.330	0.91
α_*P*_	This work	42	0.146	0.210	0.82
	2016H66	6	0.171	0.148	0.91
	GAFF	40	0.224	0.220	0.58
	OPLS-AA	40	0.155	0.210	0.64
κ_*T*_	This work	42	0.046	0.500	0.70
	2016H66	6	0.276	0.279	0.71
	GAFF	40	0.054	0.150	0.77
	OPLS-AA	40	−0.016	0.130	0.78
ε	This work	42	−4.523	5.650	0.65
	2016H66	6	−2.217	2.515	0.89
	GAFF	29	−4.254	2.740	0.97
	OPLS-AA	33	−4.564	5.600	0.72

Regarding the targeted properties, our calibration set yielded the equations *y* = 0.9118*x* + 0.1001 for density, *y* = 1.0699*x* − 1.6491 for enthalpy of vaporization and *y* = 0.8676*x* + 0.8929 for free energy of solvation, with correlation coefficients of *R* = 0.92, *R* = 0.96, and *R* = 0.89, respectively. In terms of average deviation (AVED), our calibration set overestimates ρ in 0.008 g/cm^3^, Δ*H*_*vap*_ in 1.51 kJ/mol and underestimates Δ*G*_*hyd*_ in 3.35 kJ/mol. Without the outliers, the AVED for Δ*G*_*hyd*_ improves to 2.83 kJ/mol.

Non-targeted properties were calculated to evaluate how they behaved in our simulations. Linear regressions yielded equations *y* = 0.93825*x* + 0.1406 for α_*P*_ (R = 0.82), *y* = +0.90079*x* − 0.0140 for κ_*T*_ (*R* = 0.70), *y* = 0.2581*x* + 1.8961 for ε (*R* = 0.65), and *y* = 0.8989*x* + 100.5 for *Cp*_*cla*_ (*R* = 0.77). In terms of AVED, α_*P*_ is overestimated in 0.14 10^−3^/K and κ_*T*_ is overestimated in 0.0465 1/GPa. As expected (Caleman et al., 2012; Horta et al., 2016), ε is poorly described due to the lack of polarization effects, resulting in a underestimation of −4.52 in the dielectric constant. On other hand, *Cp*_*cla*_ was overestimated by 88.2 J/mol × K, a behavior aligned with recent works in literature (Caleman et al., 2012; Horta et al., 2016). Individual AVED and absolute errors can be found in Tables S4, S5 in Supplementary Material, along with experimental properties in Table S3.

### 3.3. Interactions in water

In order to quantitatively evaluate the behavior of heteroaromatic rings in water and their interactions with the aqueous surrounding, some properties were calculated throughout 250 ns of simulation. From these calculations, we were capable to assess the average H-bond (*Aver*_*HB*_) of each heteroatom along with its residence time (τ_*HB*_), lifetime (*lifetime*_*HB*_), the free-energy of breakage of a H-bond (Δ*G*_*HB*_), and the percentage of simulation time that a given heteroatom was involved in, at least, one H-bond (*Percent*). We were also capable to obtain the optimal binding distance between an heteratom and water (*OBD*_*HB*_), along with the coordination number (*CN*_*HB*_) at the *OBD*_*HB*_ and the average orientation of water molecules surrounding the heteroatom. These data are compiled in Tables 4, 5.

**Table 4:** Properties of heteroaromatic rings in water. Average H-bonds (*Aver*_*HB*_), H-bond residence time (τ_*HB*_) is ps, H-bond lifetime (*lifetime*_*HB*_) in 1/ps, free-energy of H-bond breakage (Δ*G*_*HB*_) in kJ/mol, percentage of simulation with at least one formed H-bond (*Percent*.), coordination number of water (*CN*), optimal binding distance with water (*OBD*_*HB*_) in nm, and overall water orientation around the heteroatom (*Orientation*).

**Molecule**	**Atom**	***Aver*_*HB*_**	**τ_*HB*_**	***lifetime*_*HB*_**	***ΔG*_*HB*_**	***Percent***	**CN**	***OBD*_*HB*_**	***Orientation***
Water	**Ow**	1.73 ± 0.62	2.11 ± 0.02	0.47 ± 0.00	6.38 ± 0.03	98.58	4.11 ± 2.83	0.18 ± 0.00	Undefined
	**OH**^**1**^	0.87 ± 0.35	1.80 ± 0.03	0.55 ± 0.01	5.98 ± 0.05	86.25	4.11 ± 2.83	0.18 ± 0.00	O-oriented
	**OH**^**2**^	0.86 ± 0.35	1.83 ± 0.03	0.54 ± 0.01	6.03 ± 0.04	86.07	4.11 ± 2.83	0.18 ± 0.00	O-oriented
Phenol	**O**	1.10 ± 0.62	1.61 ± 0.03	0.62 ± 0.01	5.70 ± 0.04	85.96	1.46 ± 1.03	0.18 ± 0.00	Undefined
	**OH**	0.96 ± 0.20	9.49 ± 0.18	0.11 ± 0.00	10.11 ± 0.05	96.04	0.90 ± 0.01	0.17 ± 0.00	O-oriented
Phenylmethanol	**O**	1.42 ± 0.58	2.58 ± 0.03	0.39 ± 0.00	6.88 ± 0.02	96.51	2.68 ± 1.59	0.18 ± 0.00	Undefined
	**OH**	0.95 ± 0.24	5.37 ± 0.06	0.19 ± 0.00	8.70 ± 0.03	94.25	1.13 ± 0.01	0.17 ± 0.00	O-oriented
2-methylphenol	**O**	1.04 ± 0.59	1.88 ± 0.04	0.53 ± 0.01	6.09 ± 0.05	84.80	1.05 ± 0.00	0.18 ± 0.00	Undefined
	**OH**	0.95 ± 0.23	9.46 ± 0.17	0.11 ± 0.00	10.10 ± 0.04	94.53	0.87 ± 0.01	0.17 ± 0.00	O-oriented
3-methylphenol	**O**	1.08 ± 0.61	1.74 ± 0.02	0.58 ± 0.01	5.90 ± 0.03	85.83	1.43 ± 1.00	0.18 ± 0.00	Undefined
	**OH**	0.96 ± 0.19	10.12 ± 0.19	0.10 ± 0.00	10.27 ± 0.05	96.30	0.90 ± 0.01	0.17 ± 0.00	O-oriented
4-methylphenol	**O**	1.08 ± 0.61	1.73 ± 0.02	0.58 ± 0.01	5.89 ± 0.03	85.70	1.10 ± 0.01	0.18 ± 0.00	Undefined
	**OH**	0.96 ± 0.20	10.00 ± 0.21	0.10 ± 0.00	10.24 ± 0.05	96.21	0.90 ± 0.01	0.17 ± 0.00	O-oriented
Benzenethiol	**S**	0.67 ± 0.65	0.38 ± 0.01	2.63 ± 0.05	2.13 ± 0.04	57.29	0.81 ± 0.17	0.23 ± 0.00	Undefined
	**SH**	0.77 ± 0.43	1.00 ± 0.02	1.00 ± 0.02	4.52 ± 0.05	76.38	2.08 ± 0.02	0.23 ± 0.00	O-oriented
Aniline	**N**	0.93 ± 0.58	1.64 ± 0.02	0.61 ± 0.01	5.75 ± 0.03	79.89	1.01 ± 0.01	0.19 ± 0.00	Undefined
	**NH**^**1**^	0.63 ± 0.49	1.15 ± 0.03	0.87 ± 0.02	4.87 ± 0.06	62.48	1.25 ± 0.38	0.22 ± 0.00	O-oriented
	**NH**^**2**^	0.63 ± 0.50	0.99 ± 0.02	1.01 ± 0.02	4.51 ± 0.04	62.05	1.39 ± 0.25	0.23 ± 0.00	O-oriented
2-chloroaniline	**N**	0.86 ± 0.50	2.29 ± 0.04	0.44 ± 0.01	6.59 ± 0.05	79.39	0.92 ± 0.00	0.19 ± 0.00	Undefined
	**NH**^**1**^	0.51 ± 0.51	1.00 ± 0.03	1.00 ± 0.03	4.53 ± 0.06	50.60	1.33 ± 0.14	0.23 ± 0.00	O-oriented
	**NH**^**2**^	0.56 ± 0.51	0.87 ± 0.03	1.15 ± 0.04	4.18 ± 0.09	55.20	3.82 ± 5.29	0.23 ± 0.01	O-oriented
	**Cl**	0.24 ± 0.45	0.32 ± 0.08	3.26 ± 0.68	1.66 ± 0.56	22.67	18.94 ± 10.05	0.36 ± 0.00	Undefined
Pyridine	**N**	1.41 ± 0.71	1.33 ± 0.02	0.75 ± 0.01	5.24 ± 0.03	91.46	1.59 ± 0.01	0.20 ± 0.00	Undefined
Pyrimidine	**N**^**1**^	1.06 ± 0.68	0.91 ± 0.02	1.10 ± 0.02	4.30 ± 0.05	80.71	1.23 ± 0.01	0.20 ± 0.00	Undefined
	**N**^**2**^	0.98 ± 0.68	0.81 ± 0.02	1.24 ± 0.03	4.00 ± 0.06	76.96	1.17 ± 0.01	0.20 ± 0.00	Undefined
2-methylpyridine	**N**	1.52 ± 0.70	1.74 ± 0.04	0.57 ± 0.01	5.90 ± 0.06	93.96	1.68 ± 0.00	0.20 ± 0.00	Undefined
3-methylpyridine	**N**	1.43 ± 0.71	1.34 ± 0.04	0.74 ± 0.02	5.26 ± 0.07	91.65	1.61 ± 0.01	0.20 ± 0.00	Undefined
4-methylpyridine	**N**	1.46 ± 0.71	1.44 ± 0.04	0.69 ± 0.02	5.44 ± 0.07	92.57	1.62 ± 0.01	0.20 ± 0.00	Undefined
2,4,6-trimethylpyridine	**N**	0.36 ± 0.53	0.36 ± 0.04	2.79 ± 0.31	2.00 ± 0.28	33.67	24.48 ± 3.47	0.42 ± 0.09	Undefined
Quinoline	**N**	1.64 ± 0.68	2.00 ± 0.05	0.50 ± 0.01	6.25 ± 0.06	96.10	1.78 ± 0.01	0.19 ± 0.00	Undefined
Isoquinoline	**N**	1.26 ± 0.68	1.22 ± 0.04	0.82 ± 0.02	5.02 ± 0.07	88.67	1.43 ± 0.01	0.20 ± 0.00	Undefined
Benzonitrile	**N**	1.63 ± 0.72	1.30 ± 0.01	0.77 ± 0.01	5.17 ± 0.02	95.50	1.88 ± 0.01	0.19 ± 0.00	Undefined
Furan	**O**	0.42 ± 0.57	0.29 ± 0.01	3.41 ± 0.07	1.49 ± 0.05	37.99	31.54 ± 2.60	0.46 ± 0.01	Undefined
Tiophene	**S**	0.15 ± 0.37	0.25 ± 0.03	4.07 ± 0.49	1.07 ± 0.32	14.03	18.25 ± 5.37	0.37 ± 0.00	Undefined
Pyrrole	**NH**	0.92 ± 0.29	3.80 ± 0.06	0.26 ± 0.00	7.84 ± 0.04	91.73	0.38 ± 0.00	0.18 ± 0.00	O-oriented
	**N**	0.74 ± 0.67	1.33 ± 0.03	0.75 ± 0.02	5.23 ± 0.06	60.90	0.62 ± 0.01	0.23 ± 0.00	Undefined
Fluorobenzene	**F**^**1**^	0.30 ± 0.49	0.30 ± 0.03	3.35 ± 0.32	1.54 ± 0.24	27.84	13.84 ± 5.27	0.36 ± 0.01	Undefined
1,2-difluorobenzene	**F**^**1**^	0.24 ± 0.45	0.33 ± 0.07	3.14 ± 0.61	1.74 ± 0.49	22.91	12.15 ± 2.45	0.37 ± 0.01	Undefined
	**F**^**2**^	0.24 ± 0.45	0.34 ± 0.07	3.08 ± 0.64	1.79 ± 0.51	22.90	13.31 ± 3.02	0.37 ± 0.01	Undefined
1,3-difluorobenzene	**F**^**1**^	0.23 ± 0.45	0.36 ± 0.10	2.91 ± 0.58	1.94 ± 0.59	22.23	14.99 ± 5.60	0.36 ± 0.01	Undefined
	**F**^**3**^	0.23 ± 0.45	0.32 ± 0.04	3.20 ± 0.36	1.66 ± 0.29	22.22	11.70 ± 1.33	0.36 ± 0.00	Undefined
1,2,3,4-tetrafluorobenzene	**F**^**1**^	0.17 ± 0.38	0.36 ± 0.08	2.88 ± 0.61	1.97 ± 0.53	16.08	12.99 ± 3.94	0.37 ± 0.01	Undefined
	**F**^**2**^	0.18 ± 0.40	0.42 ± 0.15	2.66 ± 0.78	2.22 ± 0.79	17.44	12.72 ± 2.45	0.37 ± 0.01	Undefined
	**F**^**3**^	0.18 ± 0.40	0.33 ± 0.04	3.11 ± 0.45	1.74 ± 0.34	17.31	13.89 ± 3.36	0.37 ± 0.01	Undefined
	**F**^**4**^	0.16 ± 0.38	0.43 ± 0.21	2.72 ± 0.83	2.20 ± 0.98	16.05	11.25 ± 1.36	0.36 ± 0.00	Undefined
1,2,3,5-tetrafluorobenzene	**F**^**1**^	0.17 ± 0.39	0.37 ± 0.10	2.84 ± 0.64	2.01 ± 0.61	16.51	12.34 ± 3.05	0.36 ± 0.01	Undefined
	**F**^**2**^	0.16 ± 0.37	0.48 ± 0.25	2.47 ± 0.82	2.47 ± 1.03	15.19	12.79 ± 3.60	0.37 ± 0.01	Undefined
	**F**^**3**^	0.17 ± 0.39	0.44 ± 0.10	2.40 ± 0.53	2.42 ± 0.56	16.59	14.91 ± 5.13	0.37 ± 0.01	Undefined
	**F**^**5**^	0.21 ± 0.43	0.33 ± 0.06	3.17 ± 0.54	1.70 ± 0.44	20.59	11.51 ± 1.36	0.36 ± 0.01	Undefined
Trifluoromethylbenzene	**F**^**1**^	0.10 ± 0.30	1.64 ± 1.99	1.26 ± 0.91	4.66 ± 2.10	9.56	14.84 ± 3.37	0.39 ± 0.02	Undefined
	**F**^**2**^	0.10 ± 0.30	0.64 ± 0.37	1.95 ± 0.73	3.10 ± 1.16	9.66	15.37 ± 3.44	0.40 ± 0.02	Undefined
	**F**^**3**^	0.10 ± 0.30	2.82 ± 5.74	1.05 ± 0.41	5.00 ± 2.38	9.54	14.94 ± 3.23	0.40 ± 0.02	Undefined
1-chloronaphthalene	**Cl**	0.37 ± 0.55	0.28 ± 0.02	3.55 ± 0.21	1.39 ± 0.15	33.96	15.53 ± 7.71	0.36 ± 0.00	Undefined
1-phenylethanone	**O**	0.87 ± 0.66	0.56 ± 0.01	1.79 ± 0.04	3.09 ± 0.05	71.14	1.08 ± 0.01	0.19 ± 0.00	Undefined
Benzaldehyde	**O**	1.03 ± 0.66	0.78 ± 0.01	1.28 ± 0.02	3.91 ± 0.04	80.79	1.22 ± 0.01	0.18 ± 0.00	Undefined
Nitrobenzene	**O**^**1**^	0.14 ± 0.36	0.43 ± 0.13	2.51 ± 0.67	2.34 ± 0.72	13.79	13.60 ± 4.08	0.38 ± 0.01	Undefined
	**O**^**2**^	0.14 ± 0.36	0.46 ± 0.15	2.37 ± 0.62	2.48 ± 0.73	13.81	16.48 ± 5.81	0.38 ± 0.01	Undefined
Methylbenzoate	**O**^**1**^	0.95 ± 0.67	0.69 ± 0.01	1.45 ± 0.02	3.62 ± 0.04	75.85	1.13 ± 0.01	0.19 ± 0.00	Undefined
	**O**^**2**^	0.17 ± 0.38	0.28 ± 0.05	3.63 ± 0.58	1.37 ± 0.43	16.40	25.27 ± 2.00	0.45 ± 0.00	Undefined
2-hydroxy-methylbenzoate	**O**	0.96 ± 0.58	1.48 ± 0.02	0.67 ± 0.01	5.51 ± 0.03	81.17	1.07 ± 0.00	0.18 ± 0.00	Undefined
	**O**^**1**^	0.94 ± 0.64	1.07 ± 0.17	0.96 ± 0.15	4.65 ± 0.39	76.43	1.07 ± 0.13	0.18 ± 0.00	Undefined
	**O**^**2**^	0.12 ± 0.33	0.36 ± 0.23	3.43 ± 1.03	1.65 ± 1.10	12.11	23.62 ± 8.28	0.33 ± 0.11	Undefined
	**OH**	0.05 ± 0.22	0.21 ± 0.04	4.83 ± 0.80	0.66 ± 0.46	5.25	0.40 ± 0.01	0.18 ± 0.01	O-oriented
Methoxybenzene	**O**	0.36 ± 0.51	0.33 ± 0.02	3.03 ± 0.14	1.78 ± 0.12	34.78	0.42 ± 0.01	0.20 ± 0.00	H-oriented
1,2-dimethoxybenzene	**O**^**1**^	0.39 ± 0.54	0.38 ± 0.02	2.62 ± 0.15	2.14 ± 0.14	36.51	20.52 ± 10.11	0.28 ± 0.08	H-oriented
	**O**^**1**^	0.39 ± 0.54	0.38 ± 0.05	2.64 ± 0.32	2.14 ± 0.32	36.49	12.48 ± 11.98	0.24 ± 0.00	H-oriented
Phenoxybenzene	**O**	0.32 ± 0.49	0.29 ± 0.02	3.40 ± 0.18	1.50 ± 0.13	31.12	5.69 ± 10.11	0.23 ± 0.01	Undefined

*Colors represent different functional groups: red for oxygen, blue for nitrogen, orange for sulfur and green for halogen containing groups*.

**Table 5:** Properties of heteroaromatic rings in water. Average H-bonds (*Aver*_*HB*_), H-bond residence time (τ_*HB*_) is ps, H-bond lifetime (*lifetime*_*HB*_) in 1/ps, free-energy of H-bond breakage (Δ*G*_*HB*_) in kJ/mol, percentage of simulation with at least one formed H-bond (*Percent*.), coordination number of water (*CN*), optimal binding distance with water (*OBD*_*HB*_) in nm, and overall water orientation around the heteroatom (*Orientation*).

**Molecule**	**Atom**	***Aver*_*HB*_**	**τ_*HB*_**	***lifetime*_*HB*_**	***ΔG*_*HB*_**	***Percent***	***CN***	***OBD*_*HB*_**	***Orientation***
Water	**Ow**	1.73 ± 0.62	2.11 ± 0.02	0.47 ± 0.00	6.38 ± 0.03	98.58	4.11 ± 2.83	0.18 ± 0.00	Undefined
	**OH1**	0.87 ± 0.35	1.80 ± 0.03	0.55 ± 0.01	5.98 ± 0.05	86.25	4.11 ± 2.83	0.18 ± 0.00	O-oriented
	**OH2**	0.86 ± 0.35	1.83 ± 0.03	0.54 ± 0.01	6.03 ± 0.04	86.07	4.11 ± 2.83	0.18 ± 0.00	O-oriented
Imidazole	**N**^**1**^	0.08 ± 0.27	0.33 ± 0.09	3.27 ± 0.86	1.68 ± 0.69	7.58	26.01 ± 7.18	0.41 ± 0.01	Undefined
	**N**^**1**^**H**	0.56 ± 0.52	0.35 ± 0.00	2.89 ± 0.04	1.90 ± 0.03	55.10	34.04 ± 1.08	0.45 ± 0.02	Undefined
	**N**^**3**^	1.30 ± 0.72	1.01 ± 0.01	0.99 ± 0.00	4.56 ± 0.01	87.72	7.56 ± 12.01	0.20 ± 0.00	Undefined
Thiazole	**S**^**1**^	0.04 ± 0.20	–	–	–	4.21	16.56 ± 5.11	0.38 ± 0.01	Undefined
	**N**^**3**^	0.53 ± 0.60	0.37 ± 0.01	2.73 ± 0.08	2.04 ± 0.07	47.16	0.62 ± 0.11	0.22 ± 0.00	Undefined
Benzopyrrole	**N**^**1**^	0.09 ± 0.29	0.28 ± 0.05	3.68 ± 0.49	1.32 ± 0.37	8.60	15.88 ± 2.99	0.38 ± 0.00	Undefined
	**N**^**1**^**H**	0.63 ± 0.51	0.66 ± 0.01	1.50 ± 0.03	3.51 ± 0.04	61.63	21.05 ± 1.45	0.41 ± 0.01	Undefined
Tetrazole	**N**^**4**^	0.87 ± 0.69	0.54 ± 0.01	1.85 ± 0.03	3.00 ± 0.04	69.85	1.17 ± 0.02	0.21 ± 0.00	Undefined
	**N**^**3**^	0.89 ± 0.74	0.53 ± 0.02	1.87 ± 0.06	2.97 ± 0.08	68.12	1.09 ± 0.32	0.23 ± 0.00	Undefined
	**N**^**2**^	0.31 ± 0.51	0.27 ± 0.02	3.75 ± 0.22	1.26 ± 0.15	29.37	26.38 ± 7.22	0.41 ± 0.00	Undefined
	**N**^**1**^	0.02 ± 0.14	–	–	–	1.95	21.88 ± 2.06	0.41 ± 0.01	Undefined
	**N**^**1**^**H**	0.00 ± 0.00	–	–	–	0.00	0.50 ± 0.03	0.24 ± 0.00	O-oriented
Benzeimidazole	**N**^**1**^	0.06 ± 0.23	0.21 ± 0.03	4.87 ± 0.67	0.62 ± 0.34	5.53	16.49 ± 2.30	0.40 ± 0.01	Undefined
	**N**^**1**^**H**	0.78 ± 0.44	1.21 ± 0.02	0.82 ± 0.01	5.01 ± 0.03	77.23	32.26 ± 2.16	0.46 ± 0.01	Undefined
	**N**^**3**^	1.08 ± 0.71	0.86 ± 0.01	1.16 ± 0.02	4.16 ± 0.04	80.28	1.32 ± 0.01	0.20 ± 0.00	Undefined
7,8-dihydro-1H-purine	**N**^**6**^**H**	0.42 ± 0.51	0.34 ± 0.01	2.91 ± 0.09	1.88 ± 0.08	41.30	30.55 ± 3.92	0.46 ± 0.01	Undefined
	**N**^**1**^	0.04 ± 0.19	0.20 ± 0.02	5.21 ± 0.66	0.46 ± 0.31	3.73	16.89 ± 12.64	0.28 ± 0.00	O-oriented
	**N**^**6**^	0.04 ± 0.19	–	–	–	3.70	28.25 ± 2.59	0.46 ± 0.01	Undefined
	**N**^**4**^	0.33 ± 0.51	0.37 ± 0.04	2.76 ± 0.31	2.02 ± 0.29	31.14	24.55 ± 2.24	0.45 ± 0.01	Undefined
	**N**^**1**^**H**	0.96 ± 0.21	6.49 ± 0.11	0.15 ± 0.00	9.16 ± 0.04	95.83	0.79 ± 0.65	0.18 ± 0.00	O-oriented
	**N**^**3**^	1.83 ± 0.70	1.97 ± 0.09	0.51 ± 0.02	6.20 ± 0.11	97.65	3.38 ± 1.14	0.20 ± 0.00	Undefined
1,2,4 - Triazole	**N**^**2**^	1.50 ± 0.72	1.53 ± 0.04	0.65 ± 0.02	5.59 ± 0.06	92.83	1.70 ± 0.00	0.20 ± 0.00	Undefined
	**N**^**1**^	0.66 ± 0.66	0.83 ± 0.02	1.20 ± 0.04	4.07 ± 0.07	55.78	2.10 ± 3.51	0.22 ± 0.00	Undefined
	**N**^**1**^**H**	0.98 ± 0.15	11.47 ± 0.25	0.09 ± 0.00	10.58 ± 0.05	97.94	3.76 ± 0.00	0.25 ± 0.03	O-oriented
	**N**^**4**^	0.83 ± 0.68	0.61 ± 0.01	1.63 ± 0.02	3.32 ± 0.04	67.99	0.97 ± 0.01	0.20 ± 0.00	Undefined
Quinazoline	**N**^**1**^	0.64 ± 0.63	0.49 ± 0.02	2.04 ± 0.07	2.76 ± 0.09	56.17	0.71 ± 0.21	0.21 ± 0.00	Undefined
	**N**^**3**^	0.43 ± 0.56	0.31 ± 0.01	3.19 ± 0.05	1.65 ± 0.04	39.34	24.86 ± 3.16	0.28 ± 0.08	H-oriented
1H-pyrimidin-2-one	**O**^**2**^	1.20 ± 0.72	0.79 ± 0.01	1.27 ± 0.02	3.93 ± 0.03	84.99	1.42 ± 0.02	0.20 ± 0.00	Undefined
	**N**^**1**^**H**	0.41 ± 0.51	0.33 ± 0.01	3.07 ± 0.13	1.75 ± 0.10	39.74	24.01 ± 4.58	0.44 ± 0.02	Undefined
	**N**^**1**^	0.01 ± 0.09	–	–	–	0.73	19.00 ± 2.14	0.41 ± 0.00	Undefined
	**N**^**3**^	0.89 ± 0.62	0.84 ± 0.00	1.19 ± 0.01	4.11 ± 0.01	75.00	1.05 ± 0.00	0.20 ± 0.00	Undefined
4-quinolone	**O**^**4**^	1.74 ± 0.74	1.39 ± 0.02	0.72 ± 0.01	5.35 ± 0.04	96.17	3.85 ± 1.52	0.19 ± 0.00	Undefined
	**N**^**1**^	0.01 ± 0.10	–	–	–	1.03	23.69 ± 2.01	0.47 ± 0.01	Undefined
	**N**^**1**^**H**	0.66 ± 0.49	0.80 ± 0.02	1.26 ± 0.04	3.96 ± 0.07	65.61	26.90 ± 2.65	0.47 ± 0.01	Undefined
Isoxazole	**O**^**1**^	0.59 ± 0.62	0.36 ± 0.00	2.82 ± 0.02	1.96 ± 0.02	52.43	7.34 ± 13.27	0.22 ± 0.01	Undefined
	**N**^**2**^	0.60 ± 0.63	0.35 ± 0.01	2.86 ± 0.05	1.92 ± 0.04	52.00	0.77 ± 0.04	0.24 ± 0.00	H-oriented
Uracil	**N**^**3**^	0.02 ± 0.16	1.99 ± 1.45	1.04 ± 0.90	5.38 ± 2.21	2.48	17.32 ± 1.01	0.42 ± 0.01	Undefined
	**N**^**3**^**H**	0.33 ± 0.49	0.29 ± 0.01	3.47 ± 0.10	1.45 ± 0.07	31.98	25.64 ± 8.27	0.43 ± 0.02	Undefined
	**O**^**2**^	0.39 ± 0.54	0.28 ± 0.01	3.61 ± 0.13	1.35 ± 0.09	36.17	19.18 ± 8.50	0.37 ± 0.01	Undefined
	**O**^**4**^	1.24 ± 0.71	0.87 ± 0.01	1.15 ± 0.01	4.19 ± 0.02	86.66	4.08 ± 5.14	0.20 ± 0.00	Undefined
	**N**^**1**^	0.01 ± 0.07	–	–	–	0.54	29.81 ± 2.46	0.46 ± 0.01	Undefined
	**N**^**1**^**H**	0.45 ± 0.52	0.37 ± 0.01	2.67 ± 0.07	2.09 ± 0.06	44.10	30.23 ± 1.97	0.47 ± 0.01	Undefined
Pyrazole	**N**^**1**^**H**	0.00 ± 0.00	−	−	−	0.00	18.32 ± 2.00	0.40 ± 0.01	Undefined
	**N**^**1**^	0.00 ± 0.00	–	–	–	0.00	17.76 ± 1.63	0.40 ± 0.00	Undefined
	**N**^**2**^	0.72 ± 0.66	0.44 ± 0.01	2.29 ± 0.07	2.48 ± 0.08	60.69	0.96 ± 0.03	0.21 ± 0.00	Undefined
Pyrazine	**N**^**1**^	1.15 ± 0.66	1.15 ± 0.03	0.87 ± 0.02	4.88 ± 0.07	85.83	6.55 ± 10.50	0.19 ± 0.00	Undefined
	**N**^**4**^	1.15 ± 0.65	1.15 ± 0.02	0.87 ± 0.02	4.86 ± 0.05	85.87	6.66 ± 10.74	0.19 ± 0.00	Undefined
1,8-naphthyridin-4(1H)-one	**O**^**4**^	1.13 ± 0.73	0.71 ± 0.02	1.41 ± 0.04	3.68 ± 0.07	81.51	1.44 ± 0.02	0.20 ± 0.00	Undefined
	**N**^**8**^	0.28 ± 0.48	0.28 ± 0.02	3.57 ± 0.28	1.38 ± 0.19	26.76	25.87 ± 2.34	0.45 ± 0.01	Undefined
	**N**^**1**^	0.05 ± 0.23	0.40 ± 0.14	2.81 ± 0.93	2.10 ± 0.84	5.34	22.03 ± 2.09	0.44 ± 0.01	Undefined
	**N**^**1**^**H**	0.40 ± 0.51	0.34 ± 0.02	2.99 ± 0.20	1.82 ± 0.17	39.32	26.16 ± 2.29	0.45 ± 0.01	Undefined
Xanthine	**N**^**1**^**H**	0.40 ± 0.51	0.38 ± 0.01	2.66 ± 0.10	2.10 ± 0.09	39.37	28.47 ± 4.09	0.46 ± 0.02	Undefined
	**O**^**2**^	0.52 ± 0.60	0.32 ± 0.03	3.13 ± 0.23	1.71 ± 0.19	46.33	17.09 ± 8.14	0.37 ± 0.01	Undefined
	**N**^**7**^	0.02 ± 0.12	–	–	–	1.51	25.71 ± 2.69	0.45 ± 0.02	Undefined
	**N**^**7**^**H**	0.47 ± 0.52	0.43 ± 0.01	2.33 ± 0.08	2.43 ± 0.08	46.50	26.50 ± 2.03	0.46 ± 0.01	Undefined
	**N**^**1**^	0.02 ± 0.15	–	–	–	2.33	21.48 ± 4.05	0.44 ± 0.01	Undefined
	**N**^**3**^	0.03 ± 0.17	–	–	–	3.09	19.25 ± 5.54	0.41 ± 0.01	Undefined
	**O**^**6**^	0.46 ± 0.57	0.31 ± 0.01	3.25 ± 0.06	1.61 ± 0.05	42.64	8.82 ± 4.23	0.33 ± 0.06	Undefined
	**N**^**9**^	0.28 ± 0.47	0.28 ± 0.03	3.61 ± 0.36	1.36 ± 0.25	27.40	26.67 ± 2.40	0.46 ± 0.01	Undefined
1,2-dihydro-3H-1,2,4-triazol-3-one	**N**^**1**^**H**	0.95 ± 0.24	4.50 ± 0.09	0.22 ± 0.00	8.25 ± 0.05	94.28	1.32 ± 1.05	0.17 ± 0.00	O-oriented
	**N**^**2**^**H**	0.48 ± 0.52	0.37 ± 0.01	2.72 ± 0.05	2.04 ± 0.05	46.54	21.76 ± 2.73	0.38 ± 0.00	Undefined
	**N**^**4**^	1.21 ± 0.67	1.11 ± 0.01	0.90 ± 0.01	4.80 ± 0.03	87.18	1.39 ± 0.00	0.20 ± 0.00	Undefined
	**O**^**3**^	1.26 ± 0.76	0.79 ± 0.01	1.27 ± 0.01	3.93 ± 0.02	85.27	1.54 ± 0.02	0.20 ± 0.00	Undefined
	**N**^**1**^	0.02 ± 0.13	–	–	–	1.75	17.03 ± 9.51	0.28 ± 0.00	O-oriented
	**N**^**2**^	0.03 ± 0.16	–	–	–	2.52	27.49 ± 5.89	0.38 ± 0.00	Undefined
1,3,4 - Thiadiazole	**S**^**1**^	0.02 ± 0.15	–	–	–	2.32	19.74 ± 5.86	0.39 ± 0.01	Undefined
	**N**^**3**^	1.33 ± 0.73	1.17 ± 0.03	0.86 ± 0.02	4.91 ± 0.05	88.26	18.35 ± 13.63	0.21 ± 0.00	Undefined
	**N**^**4**^	1.34 ± 0.73	1.16 ± 0.01	0.86 ± 0.01	4.89 ± 0.02	88.35	1.70 ± 0.02	0.21 ± 0.00	Undefined
Indoxazine	**N**^**2**^	0.69 ± 0.65	0.45 ± 0.00	2.20 ± 0.02	2.57 ± 0.02	59.11	0.94 ± 0.02	0.22 ± 0.00	Undefined
	**O**^**1**^	0.74 ± 0.66	0.48 ± 0.01	2.08 ± 0.05	2.72 ± 0.06	62.08	0.84 ± 0.21	0.21 ± 0.00	Undefined
3,9-dihydro-6H-purin-6-one	**N**^**1**^**H**	0.46 ± 0.52	0.40 ± 0.01	2.48 ± 0.09	2.28 ± 0.09	45.20	26.74 ± 2.00	0.45 ± 0.00	Undefined
	**N**^**1**^	0.02 ± 0.14	–	–	–	1.93	22.39 ± 5.57	0.42 ± 0.01	Undefined
	**N**^**9**^	0.03 ± 0.17	0.71 ± 0.45	1.99 ± 1.06	3.23 ± 1.48	3.05	20.26 ± 3.47	0.42 ± 0.01	Undefined
	**N**^**9**^**H**	0.47 ± 0.52	0.36 ± 0.00	2.80 ± 0.03	1.97 ± 0.03	45.60	26.51 ± 3.63	0.44 ± 0.02	Undefined
	**N**^**3**^	0.11 ± 0.32	0.47 ± 0.19	2.40 ± 0.71	2.49 ± 0.88	11.22	27.05 ± 1.99	0.46 ± 0.01	Undefined
	**O**^**6**^	1.35 ± 0.77	0.82 ± 0.03	1.21 ± 0.04	4.05 ± 0.08	87.77	1.68 ± 0.02	0.20 ± 0.00	Undefined
	**N**^**7**^	0.57 ± 0.61	0.44 ± 0.02	2.28 ± 0.11	2.49 ± 0.12	50.12	0.65 ± 0.15	0.23 ± 0.00	Undefined
Benzofuran	**O**^**1**^	0.50 ± 0.59	0.35 ± 0.01	2.90 ± 0.10	1.89 ± 0.09	44.67	23.40 ± 11.48	0.32 ± 0.11	Undefined
Indazole	**N**^**2**^	0.40 ± 0.55	0.29 ± 0.02	3.45 ± 0.20	1.46 ± 0.14	36.29	22.45 ± 4.12	0.42 ± 0.01	Undefined
	**N**^**1**^	0.17 ± 0.39	0.22 ± 0.02	4.62 ± 0.40	0.75 ± 0.22	16.72	16.16 ± 2.36	0.39 ± 0.01	Undefined
	**N**^**1**^**H**	0.55 ± 0.52	0.45 ± 0.00	2.22 ± 0.02	2.55 ± 0.02	53.47	18.47 ± 4.54	0.40 ± 0.01	Undefined
Benzothiophene	**S**^**1**^	0.14 ± 0.35	0.36 ± 0.09	2.99 ± 0.83	1.91 ± 0.67	13.13	17.61 ± 6.64	0.37 ± 0.00	Undefined
Chromone	**O**^**4**^	1.17 ± 0.73	0.74 ± 0.01	1.35 ± 0.02	3.78 ± 0.04	83.02	1.46 ± 0.01	0.20 ± 0.00	Undefined
	**O**^**1**^	0.14 ± 0.35	0.39 ± 0.12	2.81 ± 0.70	2.06 ± 0.71	13.72	25.00 ± 1.49	0.46 ± 0.01	Undefined
1,4-naphthoquinone	**O**^**4**^	0.64 ± 0.61	0.44 ± 0.01	2.27 ± 0.03	2.49 ± 0.03	56.82	0.83 ± 0.01	0.20 ± 0.00	Undefined
	**O**^**1**^	0.64 ± 0.61	0.44 ± 0.01	2.25 ± 0.08	2.52 ± 0.08	57.12	0.82 ± 0.02	0.20 ± 0.00	Undefined
1,2,3 - Triazole	**N**^**1**^**H**	0.82 ± 0.41	1.16 ± 0.02	0.86 ± 0.01	4.89 ± 0.04	80.97	14.14 ± 17.25	0.24 ± 0.10	O-oriented
	**N**^**3**^	1.11 ± 0.73	0.78 ± 0.01	1.29 ± 0.02	3.90 ± 0.03	80.38	1.47 ± 0.02	0.21 ± 0.00	Undefined
	**N**^**2**^	1.09 ± 0.74	0.75 ± 0.01	1.34 ± 0.02	3.81 ± 0.03	78.90	1.47 ± 0.00	0.21 ± 0.00	Undefined
	**N**^**1**^	0.11 ± 0.32	0.21 ± 0.00	4.73 ± 0.09	0.67 ± 0.05	11.02	16.72 ± 8.74	0.39 ± 0.03	Undefined
Pyridazine	**N**^**1**^	1.42 ± 0.76	1.25 ± 0.01	0.80 ± 0.01	5.09 ± 0.02	89.58	1.83 ± 0.00	0.21 ± 0.00	Undefined
	**N**^**2**^	1.41 ± 0.76	1.24 ± 0.03	0.81 ± 0.02	5.05 ± 0.07	89.42	1.83 ± 0.00	0.21 ± 0.00	Undefined
Triazine	**N**^**1**^	0.28 ± 0.48	0.29 ± 0.03	3.46 ± 0.38	1.47 ± 0.27	26.10	29.60 ± 0.96	0.45 ± 0.01	Undefined
	**N**^**3**^	0.27 ± 0.48	0.29 ± 0.03	3.48 ± 0.35	1.45 ± 0.25	25.94	29.63 ± 1.97	0.46 ± 0.00	Undefined
	**N**^**5**^	0.27 ± 0.48	0.28 ± 0.02	3.62 ± 0.24	1.34 ± 0.16	25.78	33.15 ± 1.59	0.45 ± 0.01	Undefined
Quinoxaline	**N**^**4**^	0.34 ± 0.51	0.30 ± 0.02	3.40 ± 0.19	1.50 ± 0.14	32.01	24.66 ± 0.64	0.37 ± 0.10	Undefined
	**N**^**1**^	0.34 ± 0.51	0.30 ± 0.02	3.34 ± 0.19	1.54 ± 0.14	31.83	25.96 ± 2.58	0.33 ± 0.11	Undefined
Oxazole	**O**^**1**^	0.34 ± 0.51	0.29 ± 0.01	3.51 ± 0.16	1.42 ± 0.12	31.80	31.43 ± 3.12	0.41 ± 0.10	Undefined
	**N**^**3**^	0.42 ± 0.56	0.29 ± 0.01	3.48 ± 0.14	1.44 ± 0.10	38.27	33.46 ± 2.32	0.43 ± 0.09	Undefined
Isothiazole	**S**^**1**^	0.05 ± 0.22	0.70 ± 0.11	1.45 ± 0.20	3.63 ± 0.36	5.11	13.50 ± 1.67	0.37 ± 0.00	Undefined
	**N**^**2**^	0.30 ± 0.49	0.29 ± 0.02	3.42 ± 0.21	1.48 ± 0.15	28.32	29.65 ± 2.44	0.46 ± 0.01	Undefined
1,3,4 - Oxadiazole	**N**^**3**^	0.96 ± 0.70	0.65 ± 0.02	1.53 ± 0.04	3.47 ± 0.07	74.33	1.32 ± 0.00	0.22 ± 0.00	Undefined
	**N**^**4**^	0.96 ± 0.70	0.64 ± 0.01	1.55 ± 0.02	3.44 ± 0.04	73.98	1.31 ± 0.01	0.21 ± 0.00	Undefined
	**O**^**1**^	0.09 ± 0.29	0.71 ± 0.42	1.88 ± 0.84	3.29 ± 1.36	8.69	29.27 ± 5.26	0.44 ± 0.02	Undefined
1,2,5 - Oxadiazole	**O**^**1**^	0.64 ± 0.66	0.36 ± 0.01	2.76 ± 0.06	2.01 ± 0.06	53.93	12.92 ± 14.89	0.28 ± 0.09	H-oriented
	**N**^**2**^	0.35 ± 0.53	0.28 ± 0.02	3.59 ± 0.20	1.37 ± 0.14	32.97	27.66 ± 1.31	0.43 ± 0.01	Undefined
	**N**^**5**^	0.36 ± 0.53	0.28 ± 0.01	3.54 ± 0.14	1.40 ± 0.10	33.18	29.19 ± 3.89	0.44 ± 0.01	Undefined
1,2,4 - Oxadiazole	**N**^**2**^	0.57 ± 0.61	0.35 ± 0.01	2.83 ± 0.04	1.95 ± 0.04	50.92	7.40 ± 13.39	0.23 ± 0.01	Undefined
	**N**^**4**^	0.57 ± 0.57	0.43 ± 0.01	2.30 ± 0.07	2.46 ± 0.07	52.94	0.69 ± 0.01	0.20 ± 0.00	Undefined
	**O**^**1**^	0.53 ± 0.59	0.34 ± 0.01	2.98 ± 0.05	1.82 ± 0.04	47.71	15.71 ± 18.47	0.33 ± 0.12	Undefined
9H-purine	**N**^**3**^	0.14 ± 0.35	0.62 ± 0.38	2.12 ± 0.88	2.95 ± 1.29	13.39	22.11 ± 1.98	0.42 ± 0.01	Undefined
	**N**^**9**^**H**	0.93 ± 0.28	3.72 ± 0.10	0.27 ± 0.01	7.79 ± 0.07	92.10	0.44 ± 0.00	0.18 ± 0.00	O-oriented
	**N**^**1**^	0.89 ± 0.64	0.82 ± 0.01	1.22 ± 0.02	4.03 ± 0.04	73.61	1.05 ± 0.01	0.20 ± 0.00	Undefined
	**N**^**9**^	0.06 ± 0.25	0.23 ± 0.03	4.47 ± 0.62	0.84 ± 0.35	6.42	3.15 ± 3.45	0.29 ± 0.00	O-oriented
	**N**^**7**^	0.43 ± 0.56	0.34 ± 0.03	2.93 ± 0.23	1.87 ± 0.20	39.57	33.93 ± 0.52	0.29 ± 0.10	H-oriented
1,3-Thiazol-2-amine	**N**^**3**^	0.33 ± 0.51	0.32 ± 0.03	3.12 ± 0.28	1.72 ± 0.22	30.55	*nan*±*nan*	0.48 ± 0.01	Undefined
	**S**^**1**^	0.04 ± 0.19	–	–	–	3.62	27.86 ± 3.96	0.45 ± 0.02	Undefined
	**N**	0.77 ± 0.53	1.33 ± 0.02	0.75 ± 0.01	5.24 ± 0.04	72	0.81 ± 0.00	0.19 ± 0.00	Undefined
	**NH**^**1**^	0.74 ± 0.46	1.29 ± 0.01	0.78 ± 0.01	5.16 ± 0.03	72.89	1.35 ± 0.38	0.21 ± 0.01	O-oriented
	**NH**^**2**^	0.73 ± 0.46	1.15 ± 0.01	0.87 ± 0.01	4.88 ± 0.03	72.43	1.20 ± 0.44	0.21 ± 0.01	O-oriented
Cytosine	**N**^**1**^	0.09 ± 0.29	0.36 ± 0.14	3.07 ± 0.78	1.86 ± 0.81	8.45	25.84 ± 1.36	0.44 ± 0.01	Undefined
	**N**^**1**^**H**	0.32 ± 0.48	0.29 ± 0.01	3.41 ± 0.06	1.48 ± 0.04	31.74	29.19 ± 2.52	0.45 ± 0.00	Undefined
	**N**	0.88 ± 0.53	2.19 ± 0.03	0.46 ± 0.01	6.47 ± 0.04	79.3	8.19 ± 5.54	0.19 ± 0.00	Undefined
	**NH**^**1**^	0.73 ± 0.46	1.81 ± 0.02	0.55 ± 0.01	6.00 ± 0.02	72.76	1.06 ± 0.48	0.21 ± 0.01	O-oriented
	**NH**^**2**^	0.70 ± 0.47	1.37 ± 0.02	0.73 ± 0.01	5.30 ± 0.04	69.18	0.87 ± 0.39	0.20 ± 0.00	O-oriented
	**O**^**1**^	1.20 ± 0.79	0.69 ± 0.02	1.44 ± 0.04	3.62 ± 0.07	81.29	1.40 ± 0.39	0.21 ± 0.00	Undefined
	**N**^**3**^	0.93 ± 0.73	0.70 ± 0.05	1.44 ± 0.10	3.63 ± 0.18	71.11	1.34 ± 0.32	0.23 ± 0.00	Undefined
Adenine	**N**^**3**^	1.98 ± 0.68	3.01 ± 0.10	0.33 ± 0.01	7.26 ± 0.08	98.70	3.96 ± 1.48	0.19 ± 0.00	Undefined
	**N**^**9**^	0.18 ± 0.40	0.31 ± 0.02	3.26 ± 0.27	1.60 ± 0.20	16.99	25.32 ± 2.77	0.35 ± 0.00	Undefined
	**N**^**9**^**H**	0.30 ± 0.47	0.35 ± 0.01	2.89 ± 0.11	1.90 ± 0.09	29.54	23.64 ± 1.66	0.34 ± 0.00	Undefined
	**N**^**7**^	0.17 ± 0.39	0.30 ± 0.02	3.33 ± 0.23	1.55 ± 0.18	16.86	29.43 ± 2.90	0.45 ± 0.01	Undefined
	**N**^**1**^	0.14 ± 0.36	0.80 ± 0.49	1.96 ± 1.26	3.43 ± 1.72	13.30	21.59 ± 5.25	0.39 ± 0.00	Undefined
	**N**	0.92 ± 0.48	3.15 ± 0.08	0.32 ± 0.01	7.37 ± 0.06	84.23	4.04 ± 6.16	0.19 ± 0.00	Undefined
	**NH**^**1**^	0.68 ± 0.48	1.85 ± 0.04	0.54 ± 0.01	6.05 ± 0.05	67.41	0.66 ± 0.06	0.20 ± 0.00	O-oriented
5-methylindole	**NH**^**2**^	0.70 ± 0.47	1.50 ± 0.02	0.67 ± 0.01	5.53 ± 0.04	68.92	0.67 ± 0.03	0.20 ± 0.00	O-oriented
	**N**^**1**^	0.26 ± 0.47	0.39 ± 0.02	2.56 ± 0.11	2.20 ± 0.11	24.48	12.49 ± 0.43	0.34 ± 0.05	Undefined
	**N**^**1**^**H**	0.89 ± 0.33	3.45 ± 0.04	0.29 ± 0.00	7.60 ± 0.03	88.55	0.37 ± 0.00	0.19 ± 0.00	O-oriented
3-methyl-1H-indole	**N**^**1**^	0.18 ± 0.40	0.35 ± 0.05	2.94 ± 0.40	1.88 ± 0.34	17.01	17.42 ± 2.94	0.38 ± 0.00	Undefined
	**N**^**1**^**H**	0.71 ± 0.48	1.07 ± 0.01	0.94 ± 0.01	4.69 ± 0.03	70.01	17.37 ± 2.17	0.39 ± 0.01	Undefined
Paraxanthine	**O**^**6**^	0.58 ± 0.58	0.48 ± 0.01	2.08 ± 0.05	2.72 ± 0.06	52.88	0.76 ± 0.02	0.21 ± 0.00	Undefined
	**N**^**3**^	0.01 ± 0.10	–	–	–	1.02	18.20 ± 4.10	0.39 ± 0.01	Undefined
	**N**^**3**^**H**	0.54 ± 0.52	0.65 ± 0.02	1.55 ± 0.06	3.45 ± 0.09	52.58	18.43 ± 2.43	0.40 ± 0.01	Undefined
	**O**^**2**^	0.61 ± 0.61	0.45 ± 0.01	2.22 ± 0.05	2.55 ± 0.06	54.13	0.78 ± 0.02	0.21 ± 0.00	Undefined
	**N**^**9**^	0.79 ± 0.60	0.86 ± 0.01	1.17 ± 0.02	4.15 ± 0.04	69.54	0.95 ± 0.01	0.20 ± 0.00	Undefined
	**N**^**7**^	0.00 ± 0.05	–	–	–	0.21	28.11 ± 1.53	0.47 ± 0.00	Undefined
	**N**^**1**^	0.03 ± 0.16	–	–	–	2.66	24.93 ± 0.00	0.48 ± 0.00	Undefined
Theophylline	**N**^**7**^**H**	0.33 ± 0.49	0.33 ± 0.01	3.00 ± 0.11	1.80 ± 0.09	32.06	23.41 ± 1.68	0.44 ± 0.01	Undefined
	**O**^**6**^	0.30 ± 0.48	0.27 ± 0.01	3.73 ± 0.16	1.27 ± 0.11	28.37	13.20 ± 1.45	0.38 ± 0.01	Undefined
	**N**^**3**^	0.02 ± 0.15	–	–	−	2.41	20.89 ± 2.00	0.45 ± 0.01	Undefined
	**O**^**2**^	0.60 ± 0.62	0.40 ± 0.01	2.53 ± 0.09	2.23 ± 0.09	53.03	0.63 ± 0.21	0.22 ± 0.00	Undefined
	**N**^**9**^	0.16 ± 0.38	0.29 ± 0.03	3.44 ± 0.33	1.48 ± 0.24	15.73	27.76 ± 1.85	0.46 ± 0.01	Undefined
	**N**^**7**^	0.02 ± 0.13	–	–	–	1.78	23.16 ± 3.82	0.43 ± 0.01	Undefined
	**N**^**1**^	0.01 ± 0.12	–	–	–	1.48	25.52 ± 2.34	0.48 ± 0.01	Undefined
Theobromine	**O**^**6**^	0.26 ± 0.46	0.26 ± 0.01	3.89 ± 0.10	1.16 ± 0.06	25.11	12.25 ± 1.29	0.37 ± 0.01	Undefined
	**N**^**3**^	0.00 ± 0.06	–	–	–	0.33	27.56 ± 1.00	0.48 ± 0.01	Undefined
	**O**^**2**^	0.97 ± 0.68	0.69 ± 0.01	1.46 ± 0.01	3.59 ± 0.02	76.39	1.22 ± 0.00	0.20 ± 0.00	Undefined
	**N**^**9**^	0.10 ± 0.30	0.28 ± 0.02	3.54 ± 0.27	1.40 ± 0.19	9.65	17.11 ± 2.33	0.42 ± 0.01	Undefined
	**N**^**7**^	0.01 ± 0.10	–	–	–	1.01	25.82 ± 1.01	0.47 ± 0.01	Undefined
	**N**^**1**^**H**	0.00 ± 0.00	–	–	–	0.00	20.86 ± 1.70	0.41 ± 0.02	Undefined
	**N**^**1**^	0.03 ± 0.18	2.28 ± 1.56	0.99 ± 0.92	5.67 ± 2.35	3.16	18.46 ± 3.28	0.40 ± 0.00	Undefined
2H-tetrazol-5-thiol	**N**^**1**^**H**	0.66 ± 0.50	0.56 ± 0.01	1.79 ± 0.04	3.08 ± 0.06	65.29	24.63 ± 6.12	0.43 ± 0.01	Undefined
	**S**	0.08 ± 0.28	1.98 ± 2.59	1.55 ± 1.03	4.47 ± 2.73	8.34	24.11 ± 12.41	0.35 ± 0.00	Undefined
	**SH**	0.65 ± 0.59	0.36 ± 0.01	2.75 ± 0.08	2.02 ± 0.07	59.18	15.22 ± 7.54	0.36 ± 0.00	Undefined
	**N**^**3**^	1.05 ± 0.75	0.67 ± 0.01	1.50 ± 0.03	3.52 ± 0.06	76.69	1.44 ± 0.02	0.21 ± 0.00	Undefined
	**N**^**2**^	0.47 ± 0.58	0.34 ± 0.00	2.93 ± 0.03	1.86 ± 0.03	42.55	21.28 ± 4.89	0.40 ± 0.01	Undefined
	**N**^**1**^	0.01 ± 0.09	–	–	–	0.90	16.82 ± 4.27	0.39 ± 0.02	Undefined
	**N**^**4**^	0.54 ± 0.61	0.37 ± 0.01	2.68 ± 0.10	2.09 ± 0.09	47.48	31.80 ± 0.00	0.33 ± 0.11	Undefined
3-methylisoxazole	**O**^**1**^	0.87 ± 0.70	0.59 ± 0.01	1.71 ± 0.02	3.20 ± 0.03	69.34	0.99 ± 0.25	0.21 ± 0.00	Undefined
	**N**^**2**^	0.94 ± 0.72	0.62 ± 0.01	1.61 ± 0.02	3.35 ± 0.03	72.36	1.32 ± 0.02	0.22 ± 0.00	Undefined
5-methylisoxazole	**O**^**1**^	1.06 ± 0.71	0.79 ± 0.02	1.27 ± 0.03	3.95 ± 0.06	78.70	1.31 ± 0.02	0.20 ± 0.00	Undefined
	**N**^**2**^	1.03 ± 0.73	0.73 ± 0.01	1.37 ± 0.02	3.75 ± 0.03	76.61	1.42 ± 0.01	0.22 ± 0.00	Undefined
Methylimidazole	**N**^**3**^	1.51 ± 0.68	1.51 ± 0.03	0.66 ± 0.01	5.55 ± 0.05	94.20	11.94 ± 12.59	0.19 ± 0.00	Undefined
	**N**^**1**^	0.03 ± 0.18	0.45 ± 0.28	2.86 ± 1.15	2.20 ± 1.28	3.26	29.76 ± 1.84	0.46 ± 0.01	Undefined
2-Methylimidazole	**N**^**3**^	1.76 ± 0.68	2.28 ± 0.04	0.44 ± 0.01	6.57 ± 0.05	97.30	3.63 ± 0.94	0.19 ± 0.00	Undefined
	**N**^**1**^	0.11 ± 0.32	0.23 ± 0.02	4.45 ± 0.34	0.83 ± 0.19	10.86	15.18 ± 1.88	0.40 ± 0.01	Undefined
	**N**^**1**^**H**	0.87 ± 0.36	1.86 ± 0.02	0.54 ± 0.01	6.06 ± 0.03	86.05	0.35 ± 0.01	0.19 ± 0.00	O-oriented
Guanine	**N**^**1**^	0.00 ± 0.06	–	–	–	0.40	6.42 ± 4.70	0.27 ± 0.00	O-oriented
	**N**^**1**^**H**	0.98 ± 0.15	11.66 ± 0.29	0.09 ± 0.00	10.62 ± 0.06	97.86	2.00 ± 0.29	0.17 ± 0.00	O-oriented
	**N**^**7**^	0.98 ± 0.65	0.81 ± 0.01	1.24 ± 0.02	3.99 ± 0.03	78.40	1.19 ± 0.01	0.20 ± 0.00	Undefined
	**N**^**3**^	1.51 ± 0.64	2.33 ± 0.08	0.43 ± 0.02	6.62 ± 0.09	95.37	2.98 ± 1.13	0.19 ± 0.00	Undefined
	**N**	0.58 ± 0.57	1.16 ± 0.03	0.86 ± 0.02	4.90 ± 0.06	54.33	0.27 ± 0.02	0.19 ± 0.00	Undefined
	**NH**^**1**^	0.71 ± 0.47	2.24 ± 0.03	0.45 ± 0.01	6.53 ± 0.03	70.16	29.15 ± 1.28	0.35 ± 0.00	Undefined
	**NH**^**2**^	0.67 ± 0.48	1.62 ± 0.02	0.62 ± 0.01	5.72 ± 0.03	66.50	24.74 ± 7.45	0.34 ± 0.00	Undefined
	**O**^**6**^	1.91 ± 0.71	2.01 ± 0.02	0.50 ± 0.01	6.26 ± 0.03	98.32	3.73 ± 1.35	0.23 ± 0.05	Undefined
	**N**^**9**^	0.01 ± 0.09	–	–	–	0.76	3.81 ± 3.08	0.28 ± 0.00	O-oriented
	**N**^**9**^**H**	0.97 ± 0.19	11.16 ± 0.09	0.09 ± 0.00	10.51 ± 0.02	96.44	4.90 ± 5.94	0.17 ± 0.00	O-oriented
1-Methylindole	**N**^**1**^	0.22 ± 0.44	0.65 ± 0.20	1.80 ± 0.84	3.29 ± 0.96	20.67	26.89 ± 0.73	0.47 ± 0.00	Undefined
Chlorobenzene	**Cl**^**1**^	0.22 ± 0.44	0.34 ± 0.08	3.08 ± 0.70	1.80 ± 0.58	20.83	31.47 ± 1.09	0.36 ± 0.00	Undefined
1,2-dichlorobenzene	**Cl**^**1**^	0.17 ± 0.39	0.38 ± 0.05	2.70 ± 0.33	2.09 ± 0.32	16.31	19.59 ± 8.61	0.36 ± 0.00	Undefined
	**Cl**^**2**^	0.17 ± 0.39	0.39 ± 0.05	2.64 ± 0.38	2.14 ± 0.34	16.29	18.60 ± 8.25	0.36 ± 0.00	Undefined
1,3-dichlorobenzene	**Cl**^**1**^	0.17 ± 0.40	0.38 ± 0.08	2.78 ± 0.63	2.06 ± 0.56	16.74	30.86 ± 3.91	0.36 ± 0.00	Undefined
	**Cl**^**3**^	0.17 ± 0.39	0.37 ± 0.06	2.79 ± 0.40	2.01 ± 0.37	16.55	26.58 ± 8.67	0.36 ± 0.00	Undefined
1,2,3,4-tetrachlorobenzene	**Cl**^**4**^	0.13 ± 0.35	0.39 ± 0.12	2.75 ± 0.67	2.11 ± 0.69	12.90	25.88 ± 5.26	0.36 ± 0.00	Undefined
	**Cl**^**1**^	0.13 ± 0.35	0.43 ± 0.14	2.52 ± 0.58	2.32 ± 0.69	13.06	23.23 ± 6.96	0.37 ± 0.00	Undefined
	**Cl**^**2**^	0.11 ± 0.32	0.43 ± 0.12	2.47 ± 0.63	2.37 ± 0.66	10.45	22.80 ± 7.24	0.37 ± 0.00	Undefined
	**Cl**^**3**^	0.11 ± 0.31	0.64 ± 0.32	1.93 ± 0.80	3.14 ± 1.13	10.32	22.24 ± 7.06	0.36 ± 0.00	Undefined
1,2,3,5-tetrachlorobenzene	**Cl**^**5**^	0.16 ± 0.38	0.29 ± 0.06	3.55 ± 0.69	1.44 ± 0.49	15.90	27.66 ± 8.74	0.36 ± 0.00	Undefined
	**Cl**^**1**^	0.14 ± 0.36	0.40 ± 0.11	2.68 ± 0.57	2.15 ± 0.60	14.00	23.15 ± 9.67	0.36 ± 0.00	Undefined
	**Cl**^**2**^	0.11 ± 0.32	0.48 ± 0.25	2.51 ± 0.92	2.46 ± 1.09	10.97	22.95 ± 7.34	0.37 ± 0.00	Undefined
	**Cl**^**3**^	0.14 ± 0.36	0.79 ± 0.71	1.98 ± 0.84	3.24 ± 1.66	13.90	23.20 ± 8.70	0.36 ± 0.00	Undefined
2-pyridone	**O**^**2**^	1.55 ± 0.75	1.11 ± 0.02	0.90 ± 0.01	4.79 ± 0.04	93.28	1.82 ± 0.00	0.19 ± 0.00	Undefined
	**N**^**1**^	0.07 ± 0.27	0.24 ± 0.02	4.21 ± 0.40	0.98 ± 0.24	7.37	19.48 ± 4.86	0.43 ± 0.02	Undefined
	**N**^**1**^**H**	0.78 ± 0.43	1.40 ± 0.02	0.71 ± 0.01	5.36 ± 0.03	77.75	26.08 ± 3.66	0.44 ± 0.01	Undefined
1,3,5-triazin-2(1H)-one	**N**^**3**^	1.09 ± 0.70	1.00 ± 0.03	1.01 ± 0.03	4.52 ± 0.08	80.87	1.35 ± 0.00	0.20 ± 0.00	Undefined
	**N**^**5**^	0.11 ± 0.32	0.38 ± 0.20	3.10 ± 0.99	1.91 ± 1.04	10.86	26.48 ± 3.24	0.45 ± 0.01	Undefined
	**N**^**1**^	0.03 ± 0.17	6.76 ± 12.20	1.62 ± 1.41	5.18 ± 4.11	3.06	25.39 ± 7.62	0.43 ± 0.02	Undefined
	**N**^**1**^**H**	0.61 ± 0.51	0.55 ± 0.02	1.81 ± 0.06	3.06 ± 0.08	59.92	30.73 ± 1.51	0.46 ± 0.02	Undefined
	**O**^**2**^	0.61 ± 0.66	0.41 ± 0.02	2.45 ± 0.09	2.31 ± 0.09	51.10	28.96 ± 4.10	0.35 ± 0.00	Undefined
Phenoxazine	**O**^**5**^	0.68 ± 0.65	0.45 ± 0.01	2.23 ± 0.04	2.54 ± 0.04	58.43	0.83 ± 0.01	0.21 ± 0.00	Undefined
	**N**^**10**^**H**	0.64 ± 0.50	1.10 ± 0.03	0.91 ± 0.02	4.76 ± 0.06	62.98	23.69 ± 5.31	0.45 ± 0.02	Undefined
	**N**^**10**^	0.14 ± 0.36	0.20 ± 0.01	4.98 ± 0.25	0.55 ± 0.13	13.62	14.89 ± 2.51	0.40 ± 0.01	Undefined
7H-purine	**N**^**1**^	0.40 ± 0.55	0.31 ± 0.01	3.18 ± 0.09	1.66 ± 0.07	37.45	30.48 ± 3.09	0.32 ± 0.10	Undefined
	**N**^**7**^**H**	0.48 ± 0.52	0.35 ± 0.01	2.82 ± 0.05	1.96 ± 0.05	47.21	30.09 ± 1.79	0.46 ± 0.01	Undefined
	**N**^**3**^	0.53 ± 0.61	0.37 ± 0.02	2.68 ± 0.12	2.09 ± 0.11	46.52	28.45 ± 4.00	0.45 ± 0.02	Undefined
	**N**^**9**^	0.42 ± 0.56	0.32 ± 0.01	3.13 ± 0.08	1.70 ± 0.06	38.40	29.46 ± 1.47	0.41 ± 0.08	Undefined
	**N**^**7**^	0.02 ± 0.15	1.28 ± 0.78	1.34 ± 1.03	4.52 ± 1.88	2.34	22.55 ± 4.08	0.43 ± 0.01	Undefined
1,4-benzodioxine	**O**^**4**^	0.49 ± 0.57	0.39 ± 0.01	2.58 ± 0.07	2.18 ± 0.07	45.03	0.50 ± 0.13	0.21 ± 0.00	Undefined
	**O**^**1**^	0.49 ± 0.57	0.39 ± 0.01	2.58 ± 0.06	2.18 ± 0.06	44.98	0.57 ± 0.02	0.21 ± 0.00	Undefined

*Colors represent different functional groups: red for oxygen, blue for nitrogen, orange for sulfur and green for halogen containing groups*.

## 4. Discussion

### 4.1. Topology building strategy

The accurate description of organic compounds' chemical diversity, mainly in the context of drugs and medicinal chemistry, is a challenging task in molecular mechanics since it must be described as broadly as possible by the force field fragments. However, the most common sets of MM parameters employed in biomolecules simulations are usually centered on the monomeric constituents of biopolymers and lipids, while parameters for synthetic compounds, as well as other common non-polymeric biological molecules (e.g., natural products), must be included from specific calculations or external sets of parameters.

In this sense, a proper description of torsional terms will impact directly the dynamical behavior of these small molecules, even considering that, when evaluating ligand-receptor complexes, the influence of these terms might be mitigated due to the ligand movement restriction inside the binding pocket. Still, accommodation of flexible docking derived poses, fine tunning of induced fit, and characterization of ligands conformational induction vs. selection (with potential inferences of the entropic costs of binding) require dihedrals potentials specifically adjusted to organic compounds. Hence, new parameters were generated in this work exclusively for 15 dihedrals in aromatic rings in our calibration set (Figure 1). In general, our results revealed that our MM parameters yielded a good description of the QM torsional profile, with the exceptions of [16] tiophenol, [42] phenoxybenzene, [24] phenylmethanol, and [18] trifluoromethylbenzene. For these molecules, the distribution profile was almost evenly spread, most likely due to the low energy barrier (below 2.5 kJ/mol), indicating that transient states are commonly achieved during our simulations in SPC water model. Simulations of these particular molecules in vacuum revealed little influence of water solvation in the dihedral profile (data not shown).

In another sense, the choice of an atomic charge set for ligands can drastically impact thermodynamical binding properties such as complexation free-energy and desolvation. Therefore, we employed in this work a dipole moment based strategy to describe the Coulombic contribution using physicochemical properties of organic liquids as target. The prediction power of our strategy was compared to recent comparisons of aromatic compounds in liquid phase (Caleman et al., 2012; Horta et al., 2016) and summarized in Table 3. In general, our calibration set yielded similar or lower average deviations than benchmarks made with OPLS-AA, GAFF, and 2016H66 sets for all physicochemical properties evaluated in this work. The main difference was in terms of *Cp*_*cla*_, for which GAFF and OPLS-AA overestimate nearly 40 J/mol × K more than our parameters. Still, all four parameters sets overestimates *Cp*_*cla*_. In addition, the GROMOS53A5 force field was designed to reproduce physicochemical properties, and later on adjusted to reproduce free energy of solvation and hydration (GROMOS53A6) (Oostenbrink et al., 2004). The average deviation on density, enthalpy of vaporization and free-energy of solvation of GROMOS53A5 were 0.0389 g/cm^3^, −0.4 and 3.8 kJ/mol, respectively. These values are very similar to our results, as shown in Table 3, reiterating the quality of our parameters.

It is important to mention that the employed benchmark set was built using the same Lennard-Jones parameters used in the benzene ring of phenylalanine in GROMOS53A6. While GROMOS53A6 produces a Δ*G*_*hyd*_ = 0.0 kJ/mol for benzene (phenylalanine side-chain), our benzene parameters yield a Δ*G*_*hyd*_ = −3.4 kJ/mol, a much closer value to the experimental data (Δ*G*_*hyd*_ = −3.6 kJ/mol). Nevertheless, the AVED value reveals a underestimation for free energy of hydration in our parameter set. A possible reason is that chemical functions such as nitro, fluorine, chlorine, and aldehydic carbonyls are not commonly found in biomolecules and, therefore, the LJ parameters used in GROMOS53A6 may not be properly extrapolated to synthetic compounds. Moreover, we have tested ether oxygens LJ parameters reported in Horta et al. (2011) in our pure liquid simulations of [2]furan and [23]methoxybenzene, leading to approximately the same behavior in their respective physical-chemical properties (data not shown).

### 4.2. Properties in solution: influence of nearby substitutions in H-bonds

In order to access quantitative informations regarding how aromatic rings interact with their surroundings, we performed molecular dynamics simulations for 103 aromatic rings most commonly used in drug design, including our 42 molecules calibration set. These information are condensed in the Tables 4, 5. Simulations were carried for 250 ns to properly sample multiple events of H-bond breakages and solvation shell rearrangements.

**Figure 3:**
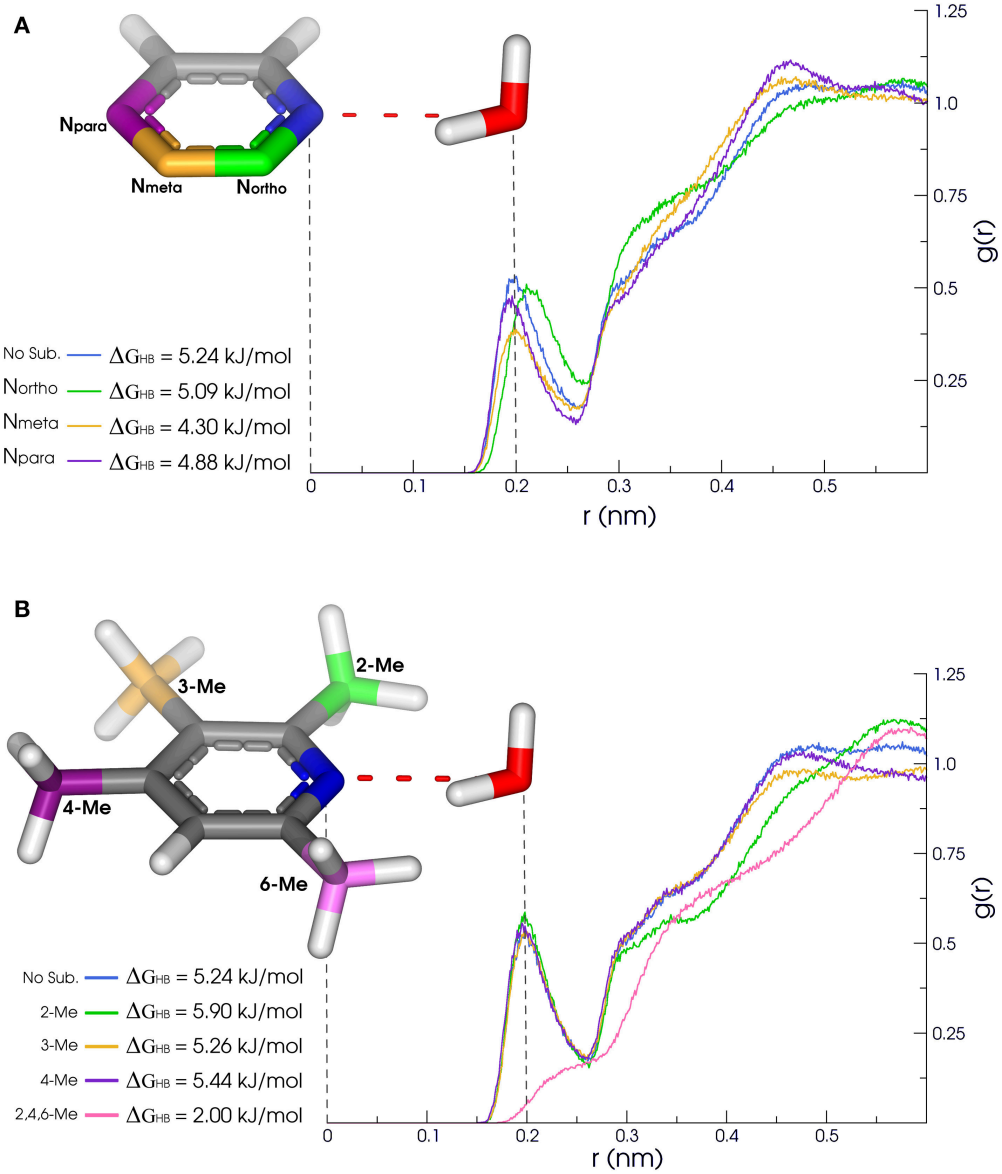
**(A)** Methyl substituitions: 2-Me (green), 3-Me (yellow), **(B)** Nearby N substitution: N_ortho_ (green), N_meta_ (yellow), 4-Me (purple) and 2,4,6-Me (pink). N_para_ (purple). Solvation properties of aromatic rings in pyridine family. Radial distribution functions (RDFs) and H-bonding strength of N^1^ (blue) are affected by substitutions in *ortho, meta*, and *para*.

Our results reveal non-obvious information about the H-bond availability and strength, as in the case of [5]pyridine/[6]pyrimidine/[56]pyrazine/[70]pyridazine/[71]triazine series (Figure 3). While exchanging a pyridine by a pyrimidine ring might lead to apparent gain of a H-bond acceptor, nitrogens of pyrimidine present a Δ*G*_*HB*_ of nearly 1 kJ/mol lower than pyridine. Moreover, the *Percent* of time with at least one formed H-bond between water and pyridine nitrogen is higher than the ones in pyrimidine. When comparing pyridine with pyrazine (an addition of another N in *para*), H-bonds are very similar, so as the second and third solvation layers. Also, acceptance capacity in pyrimidine ring is very similar to triazine, where all three nitrogens are located in *meta*. Intriguingly, values for pyridine are very similar to the ones calculated for pyridazine, with a slight increase in *OBD*_HB_ and a more compact second layer of solvation, as shown in Figure 3A. These results suggest that another nitrogen acceptor in *meta* decreases nitrogen acceptance capacity, while another nitrogen acceptor in *ortho* has low effect in H-bond capacity, but a considerable effect in the solvation layers structures. In this sense, these features can impact the binding inside receptors. Pyridazine, for example, has a larger *OBD*_HB_ than pyridine, suggesting that these molecules can occupy the binding pocket in a different manner, impacting the entropic cost of binding.

Other cases have been equally surprising, like the [39]quinoline/[40]isoquinoline. The main difference between them is the location of the acceptor nitrogen (closer to C^8^ in the quinoline fused ring). Counterintuitively, the *Aver*_*HB*_ of isoquinoline is slightly lower than for quinoline, such as the τ_*HB*_, and the Δ*G*_*HB*_ is almost 1.25 kJ/mol lower. The same properties for pyridine ring are somewhat between these values of quinoline and isoquinoline. In addition, Δ*G*_*HB*_ for [51]quinazoline and [72]quinoxaline rings are almost 3 kJ/mol lower than quinoline and isoquinoline. In this sense, quinazoline and quinoxaline would be better candidates in fragment-based drug design due to the lower energetic cost of desolvation, while maintaining the H-bond capacity inside the receptor. Another case in terms of aromatic nitrogen hydrogen bond acceptor is the [37]2,4,6-trimethylpyridine (Figure 3B). The presence of methyl groups in both *ortho* positions drastically reduces the availability of H-bonds, as shown in Figure 3, and diminish the residence time of the accepted H-bond. But the presence of only one methyl group in *ortho* appears to have a modest effect, slightly favoring the presence of H-bond in nitrogen of [19]2-methylpyridine. Moreover, the second and third solvation layers of 2- and 2,4,6-trimethylpyridine are dismantled, while the same behavior is not observed for [20]3- and [21]4-methylpyridine.

Other non-obvious events can be observed regarding H-bond donation in hydroxyls groups. In case of [12]phenol, the necessary energy to break a donated H-bond (~10 kJ/mol) is almost the double to break an accepted one (~5.70 kJ/mol), in alignment with the QM data reported by Parthasarath et al. (2005) in HF, MP2, and DFT level. And while phenol and [24]phenylmethanol might appear interchangeable during the lead optimization process, the Δ*G*_*HB*_ of accepted and donated H-bonds in the hydroxyl group is almost 1 kJ/mol higher for phenylmethanol. While targeting thermodynamics of binding during drug design, these energy costs of desolvation can play a crucial role. As expected, benzenethiol was revealed to be a poor acceptor of hydrogen bonds in our simulations, but a reasonable H-bond donator. In terms of vicinity effects, methylation in *ortho* seems to have little effect on hydroxyl groups, since the properties evaluated for the series [12]phenol/[25]2-methylphenol/[26]3-methylphenol/[27]4-methylphenol have very similar behavior.

It is well– know that halogens are widely used for drug design, and the role of halogen bonds (X-bonds) and H-bonds role have been investigated thoroughly (Rendine et al., 2011; Ford and Ho, 2016; Lin and Mackerell, 2017). In general, the H-bonding strength decreases with the halogen radius (F > Cl > Br > I), while the halogen bond strength increases (Rendine et al., 2011). In this work, we investigated how fluorine and chlorine behave as H-bond acceptors in water. In the case of [7]fluorobenzene, the Δ*G*_*HB*_ = 1.54 ± 0.24 is in accordance with a weak H-bond (Domagała et al., 2017). The other fluorinated rings in the series (1,2-, 1,3-, 1,2,3,4-, and 1,2,3,5-tetrafluorobenzene [8-11]) have similar values, varying from 1.5 to 2.2 kJ/mol. Regarding the chlorinated rings series (chlorobenzene, 1,2-, 1,3-, 1,2,3,4-, and 1,2,3,5-tetrachlorobenzene [94–98]), Δ*G*_*HB*_ ranged from 1.80 to 3.24 kJ/mol, contradicting the expected behavior. X-bonding are often poorly described in MM, since it treats atoms as a sphere with isoelectric surface and thus not describing the necessary positive potential required for such interaction. In fact, we have visually evaluated that waters surrounding fluorine and chlorine have their hydrogens oriented toward the halogens, confirming our measure of H-bonds and not X-bonds.

Regarding oxygen atoms within the aromatic ring, *Aver*_*HB*_ are generally lower than expected. It is well known that oxygens in heterocycles act as H-bond acceptor (Kaur and Khanna, 2011), but our model does not reproduce this tendency. It is important to notice that GROMOS53A6 does not have specific parameters for oxygens within aromatic rings, and LJ parameters from ethers were employed. Not surprisingly, the calculated properties for the oxygen atom in furan and benzofuran are very similar to methoxybenzene and phenoxybenzene. This result suggests that the description of the properties in aqueous solutions of aromatic rings containing oxygen might be improved by specific LJ parameters. Moreover, we have tested ether LJ parameters reported in Horta et al. (2011) for our simulations of furan and methoxybenzene in water, yielding lower *Aver*_*HB*_ and Δ*G*_*HB*_ (data not shown). The new force field parameters developed in this work can be obtained upon request.

### 4.3. Impacts in drug design

Recently, several authors have questioned the LE approach as optimization tool and its actual power to lead to high affinity compounds (Abad-Zapatero, 2007; Morgan et al., 2011; Cavalluzzi et al., 2017). Another recent review (DeGoey et al., 2017) has pointed out the emergence of approved drugs that violate Lipinski's rules of 5 and correlated them to properties such as number of aromatic rings and rotatable bonds. Freire (2009) have proposed an experimental thermodynamic approach to guide the drug design process and these results led to believe that tweaking ligand enthalpy and entropy of binding is not only experimentally possible, but also possible to predict. Therefore, the GROMOS series of force fields present an extra advantage here due to their calibration to reproduce free-energy of solvation and other thermodynamical properties.

In this sense, we have parameterized and validated a calibration set of 42 aromatic rings commonly used in drug design using thermodynamical properties in condensed phase. After, we performed a study with a larger dataset of 103 heteroaromatic rings in order to understand how these molecules interact with water and to prospect and map potential interactions with target-receptors. The water molecules probe the occurrence of hydrogen bonds, and the absence of these interactions, as well as the distance from the first solvation sphere, may probe sites for hydrophobic interactions. With these information at hand, medicinal chemists and pharmacologists may employ quantitative estimations on how each functional group may or may not interact with its target protein, as well as identify the potential influence of close chemical modifications. These properties (and a handful of others) are compiled in Tables 4, 5, and can be used as reference during lead optimization process.

The strategy employed here could be used to amplify the spectrum of drug fragments with accurate description of chemical events simulated by molecular dynamics. In addition, it can improve the description of drug-receptor complexation dynamics of other molecules of interest, molecular recognition of drugs and signal transduction mediated by conformational changes of ligands. In fact, by assessing the strength and availability of interactions between aromatic rings and water solvent, the results presented here not only offer detailed quantitative information about potential interactions that each individual aromatic ring can make with its surrounding, but also shed light upon the energetics of biological events, such as dismantling solvation shells — an important step in the ligand binding process.

## 5. Conclusions

In this work, we have successfully produced topologies for a calibration set of 42 aromatic rings using as target physicochemical properties of respective organic liquids. Our strategy revealed a very competitive prediction power when compared alongside with other force fields, while presenting a simple approach to describe aromatic rings through molecular dynamics simulations that can be easily extrapolated to other rings. In addition to that, H-bond availability and solvent accessibility are difficult and non-obvious informations to predict from bidimensional data, but still essential for medicinal chemistry purposes. Here, we have simulated in aqueous solvent more than 100 aromatic rings commonly used in drug design in order to assess dynamical chemical properties, such as average H-bonds, their lifetime, residence time and free energy of breakage. Thus, we have described a low cost approach based on molecular dynamics simulations to access valuable information that could be useful both to predict the enthalpic cost of desolvation and for interpretation of pharmacological data by a medicinal chemist or pharmacologist. Our results provide a large database of quantitative information for a total of 103 aromatic rings most commonly used in drug design that can guide medicinal chemists in future drug design efforts.

## Author contributions

MP carried out quantum calculations, molecular dynamics simulations, data analyses, and drafted the manuscript. VR contributed in the simulations protocols and manuscript draft. BG wrote in house scripts for dipole-based charge assignment and data analyses. MD contributed to manuscript draft. RL contributed to simulations protocols and manuscript draft. HV contributed to data analyses and manuscript draft.

### Conflict of interest statement

The authors declare that the research was conducted in the absence of any commercial or financial relationships that could be construed as a potential conflict of interest. The reviewer GT and handling Editor declared their shared affiliation.
