# Supplementary material for: Aromatic Rings Commonly Used in Medicinal Chemistry: Force Fields Comparison and Interactions With Water Toward the Design of New Chemical Entities
Source: Front Pharmacol. 2018 Apr 24;9:395. doi: 10.3389/fphar.2018.00395 (PMC5928326; doi:10.3389/fphar.2018.00395)
Supplement: Supplementary file 1 [file Table_1.PDF]

Table S1. Parameters used to describe the torsional profile within the working set.

Molecule Name	Code	Dihedral atoms	Phase shift	Coefficient	Multiplicity
Phenol	PHN	CD1-CG-OH-HH	0	7.541	0
			0	-9.816	2
Nitrobenzene	NBE	CD1-CG-N-O1	0	8.771	0
			0	-9.487	2
Benzenethiol	BTH	CD1-CG-SH-HH	0	0.450	4
			0	-0.953	2
Trifluoromethylbenzene	TFM	CD1-CG-CF-F1	0	0.080	6
Benzaldehyde	BNZ	CD2-CG-C-O	0	15.107	0
			0	-15.653	2
Metoxybenzene	MBO	CD1-CG-OG-CH3	0	5.941	0
			0	-11.443	2
Phenylmethanol	PHM	CD1-CG-CO-OH	0	0.816	0
			0	-1.479	2
Ethenylbenzene	ENB	CD2-CG-CB-CA	0	4.687	0
			0	-6.644	2
1-phenylethanone	1PE	CD1-CG-CO-O	0	11.226	0
			0	-12.760	2
Ethylbenzene	ETB	CD2-CG-CB-CA	0	-0.500	2
(1-methylethyl)-benzene	MEB	CD1-CG-CH-CC1	0	0.401	0
			0	-0.712	6
Aniline	ANI	CD1-CG-NG-H1	0	-1.309	2
			120	9.121	2
Methylbenzoate	MBA	CD1-CG-CO-O1	0	12.632	0
			0	22.045	1
			0	-10.542	2
Methylbenzoate	MBA	CG-CO-O2-CH3	0	23.399	0
			0	5.154	1
			0	-23.386	2
Phenoxybenzene	PBE	CD1-CG-OG-C1	0	-2.559	2
			0	0.505	6
