# Supplementary material for: Aromatic Rings Commonly Used in Medicinal Chemistry: Force Fields Comparison and Interactions With Water Toward the Design of New Chemical Entities
Source: Front Pharmacol. 2018 Apr 24;9:395. doi: 10.3389/fphar.2018.00395 (PMC5928326; doi:10.3389/fphar.2018.00395)
Supplement: Supplementary file 2 [file Table_2.PDF]

Table S2. Experimental thermodynamics properties used as reference in this work. Temperature (T) in K, density (ρ) in g/cm^3, enthalpy of vaporization (ΔH_{vap}) in kJ/mol, thermal expansion coefficient (α_P) in 10^{-3}/K, isothermal compressibility (κ_T) in 1/GPa, dielectric constant (ε), classic isobaric heat capacity (Cp_{cla}) in J/mol×K, molecular weight (MW) in g/mol and free-energy of solvation (ΔG_{hyd}) in kJ/mol.

Molecule Name	T	ρ	ΔH_{vap}	α_P	κ_T	ε	Cp_{cla}	MW	ΔG_{hyd}
Benzene	293.15	0.8765	34.08	1.23	0.96	2.27	135.70	78.112	-3.598
Pyrroline	298.15	0.9653	45.15	0.87	0.65	7.92	128.20	67.09	-20.000
Furan	298.15	0.9313	27.46	0.73	-	2.94	114.80	68.074	-
Fluorobenzene	298.15	1.0191	34.58	1.18	0.94	5.34	146.30	96.102	-3.347
1,2-fluorobenzene	298.15	1.1500	36.11	1.20	0.94*	13.59	159.00	114.093	-
1,3-fluorobenzene	298.15	1.1620	36.58	1.20	0.94*	5.06	159.10	114.093	-
1,2,3,4-fluorobenzene	298.15	1.4161	36.61	-	0.94*	-	190.06	150.074	-
1,2,3,5-fluorobenzene	298.15	1.3930	35.40	-	0.94*	-	190.19	150.074	-
Pyridine	298.15	0.9778	40.15	1.02	0.71	12.98	135.60	79.101	-19.623
Pyrimidine	298.15	1.0164	49.81	0.89	0.71*	-	133.70	80.088	-
Thiophene	298.15	1.0590	34.65	1.10	-	2.73	123.98	84.14	-5.941
Phenol	318.15	1.0545	56.32	0.80	0.92*	11.10	202.77	94.111	-27.656
Toluene	298.15	0.8619	37.99	1.07	0.92	2.37	157.20	91.139	-3.724
Quinoline	298.15	1.0900	64.10	0.73	0.44	9.00	200.00	129.159	-23.932
Isoquinoline	303.15	1.0910	59.43	0.68	0.44*	10.60	197.45	129.159	-
Nitro-benzene	298.15	1.1987	55.01	0.85	0.51	34.81	177.20	123.11	-17.238
2-chloro-aniline	293.15	1.2100	57.60	-	-	13.40	196.88	127.571	-20.543
Benzenethiol	298.15	1.0730	48.47	0.88	0.50	4.29	173.55	110.177	-10.669
2-methyl-pyridine	298.15	0.9398	42.92	0.99	0.70*	9.95	159.20	93.1262	-19.372
3-methyl-pyridine	298.15	0.9533	45.23	0.97	0.70*	11.64	159.00	93.1262	-19.958
4-methyl-pyridine	298.15	0.9503	44.81	0.96	0.70	11.96	159.00	93.1262	-20.627
Trifluoromethyl-benzene	293.15	1.1779	37.73	1.20	0.92*	9.22	188.80	146.11	-1.046
Benzonitrile	288.15	1.0093	52.14	0.83	0.92*	26.41	163.00	103.121	-17.615
Benzaldehyde	298.15	1.0436	39.60	0.25	0.23	17.40	172.00	106.122	-16.820
Methoxy-benzene	298.15	0.9894	45.00	0.95	0.69	4.22	208.60	108.138	-10.251
Phenyl-methanol	297.15	1.0419	65.59	0.69	0.92*	13.09	216.44	108.137	-27.698
2-methylphenol	308.15	1.0327	56.90	0.79	0.61*	6.44	234.03	108.137	-24.560
3-methylphenol	320.00	1.0123	60.91	0.75	0.61	10.43	233.79	108.137	-22.970
4-methyl-phenol	313.15	1.0185	63.23	0.85	-	11.21	229.41	108.137	-25.648
Ethenyl-benzene	298.15	0.9010	43.93	0.97	0.86	2.46	182.50	104.149	-5.188
1-phenyl-ethanone	298.15	1.0234	53.40	0.84	0.56	17.44	204.60	120.148	-19.163
Ethyl-benzene	298.15	0.8625	42.25	1.02	0.86	2.43	185.50	106.165	-3.305
1,2-dimethyl-benzene	298.15	0.8760	43.43	0.95	0.81	2.55	188.00	106.165	-3.766
1,2-dimethoxy-benzene	298.15	1.0820	48.38	0.93	0.69*	4.41	-	138.163	-
2,4,6-trimethyl-pyridine	295.15	0.9104	50.34	0.83	0.92*	7.81	214.00	121.179	-
(1-methylethyl)-benzene	298.15	0.8573	45.14	0.98	0.98	-	198.90	120.191	-1.255
1,2,4-trimethyl-benzene	298.15	0.8720	47.57	0.90	0.84	2.37	214.94	120.191	-3.598
1-chloro-naphthalene	298.15	1.1880	64.660	0.70	0.49	5.04	222.14	162.615	-
Aniline	298.15	1.0217	55.83	0.83	0.47	7.06	191.90	93.127	-22.970
Methyl-benzoate	298.15	1.0840	55.57	0.88	0.45	6.64	221.30	136.148	-16.401
Methyl-2-hydroxy-benzoate	298.15	1.1810	61.04	0.70	0.45*	9.47	247.51	152.147	-
Phenoxy-benzene	303.15	1.0661	58.42	0.65	-	3.65	269.87	170.207	-

** References - Experimental data extracted from Marcus (1999); Frenkel and Marsh (2003); Lide (1999); Finger et al. (1951); Findlay (1969); Yaws (2003, 2009); Hales and Townsend (1974); Abraham et al. (1990).
* Values from most similar compounds used in simulation of organic liquids.
