# Supplementary material for: Aromatic Rings Commonly Used in Medicinal Chemistry: Force Fields Comparison and Interactions With Water Toward the Design of New Chemical Entities
Source: Front Pharmacol. 2018 Apr 24;9:395. doi: 10.3389/fphar.2018.00395 (PMC5928326; doi:10.3389/fphar.2018.00395)
Supplement: Supplementary file 3 [file Table_3.PDF]

Table S3. Calculated thermodynamics properties of organic liquids extracted from Caleman *et. al* (2012)

	ρ (in g/cm^3)		ΔH_{vap} (in kJ/mol)		α_P (in 10^{-3}/K)		κ_T (in 1/GPa)		ε		Cp_{cla} (in J/mol$\times$K)	
Molecule Name	GAFF	OPLS-AA	GAFF	OPLS-AA	GAFF	OPLS-AA	GAFF	OPLS-AA	GAFF	OPLS-AA	GAFF	OPLS-AA
Benzene	-	-	-	-	-	-	-	-	-	-	-	-
Pyrroline	1.0201	0.9905	52.51	44.14	0.81	1.05	0.51	0.60	4.20	4.00	322.00	215.00
Furan	0.9660	0.9582	30.65	30.12	1.51	1.56	1.01	1.04	1.50	1.50	195.00	188.00
Fluorobenzene	0.9773	1.0214	33.46	34.45	1.60	1.20	1.26	1.01	3.30	-	247.00	230.00
1,2-fluorobenzene	1.0993	1.1193	34.05	35.27	1.53	1.67	1.26	1.4	-	11.3	249.00	272.00
1.3-fluorobenzene	1.0906	1.1071	33.78	34.03	1.36	1.75	1.25	1.44	-	3.80	249.00	260.00
1,2,3,4-fluorobenzene	1.2478	1.3483	35.45	36.85	1.77	1.52	1.84	1.39	7.20	5.30	265.00	270.00
1,2,3,5-fluorobenzene	1.2319	1.3428	33.81	37.10	1.80	1.77	1.96	1.51	-	-	276.00	289.00
Pyridine	0.9822	0.9753	41.70	41.72	1.14	1.07	0.64	0.64	-	6.70	234.00	232.00
Pyrimidine	1.1160	1.0945	50.47	49.33	1.14	1.04	0.41	0.44	8.60	8.80	222.00	222.00
Thiophene	1.0500	1.0876	34.26	39.51	1.40	1.01	0.96	0.62	-	2.60	196.00	190.00
Phenol	1.0515	1.0570	53.16	61.26	1.15	0.92	0.61	0.54	-	5.80	299.00	305.00
Toluene	0.8512	0.8720	37.39	40.02	1.62	1.49	1.06	0.88	1.10	1.20	309.00	292.00
Quinoline	1.0972	1.0864	61.13	60.40	0.78	0.90	0.49	0.48	4.00	4.00	362.00	353.00
Isoquinoline	1.0718	1.0999	62.63	74.94	0.84	0.45	0.43	0.27	4.50	2.20	340.00	319.00
Nitro-benzene	1.2331	1.1744	70.34	55.09	0.77	0.82	0.34	0.48	25.20	8.00	304.00	296.00
2-chloro-aniline	1.2392	1.2288	56.13	57.28	0.86	0.90	0.51	0.47	4.70	8.00	321.00	331.00
Benzenethiol	1.0614	1.0511	43.85	41.43	1.38	1.11	0.85	0.83	2.90	2.40	287.00	277.00
2-methyl-pyridine	0.9415	0.9480	45.34	46.07	0.96	1.14	0.72	0.63	-	5.20	275.00	281.00
3-methyl-pyridine	0.9442	0.9521	45.70	47.37	0.97	0.96	0.66	0.59	5.60	7.10	282.00	291.00
4-methyl-pyridine	0.9503	0.9484	46.06	46.40	1.29	0.95	0.67	0.60	6.60	-	281.00	283.00
Trifluoromethyl-benzene	1.1719	1.1910	41.51	38.61	1.36	1.35	1.16	1.15	6.50	-	316.00	312.00
Benzonitrile	0.9893	1.0141	53.52	54.09	1.05	0.89	0.68	0.48	16.90	6.60	294.00	248.00
Benzaldehyde	1.0369	1.0314	52.83	54.47	1.06	0.77	0.58	0.48	10.90	-	291.00	289.00
Methoxy-benzene	0.9919	0.9807	48.86	47.52	1.05	1.22	0.66	0.62	2.60	2.40	338.00	333.00
Phenyl-methanol	1.0452	1.0415	62.62	62.16	0.81	0.80	0.51	0.46	5.80	7.90	349.00	378.00
2-methyl-phenol	1.0404	1.0394	63.66	63.93	0.96	0.93	0.52	0.50	4.20	4.20	361.00	372.00
3-methyl-phenol	1.0204	1.0222	65.20	66.28	1.00	0.98	0.56	0.53	5.20	6.90	356.00	380.00
4-methyl-phenol	0.9989	1.0213	57.23	67.37	0.96	0.81	0.60	0.49	3.90	7.10	348.00	359.00
Ethenyl-benzene	0.8922	0.9121	42.43	45.02	1.14	1.18	0.88	0.74	1.10	1.00	331.00	333.00
1-phenyl-ethanone	1.0215	1.0260	58.76	61.68	1.00	0.89	0.54	0.48	11.00	6.90	392.00	360.00
Ethyl-benzene	0.8531	0.8700	42.36	44.48	1.35	1.19	0.96	0.83	1.10	1.20	372.00	366.00
1,2-dimethyl-benzene	0.8622	0.8890	42.40	46.08	1.40	1.05	0.98	0.74	1.20	1.50	362.00	360.00
1,2-dimethoxy-benzene	1.0750	1.0570	64.77	63.53	0.97	1.00	0.50	0.53	-	4.00	441.00	435.00
2,4,6-trimethyl-pyridine	0.9081	0.9295	55.78	56.74	0.92	0.89	0.68	0.59	4.00	4.40	379.00	390.00
(1-methylethyl)-benzene	0.8562	0.8739	46.90	48.71	1.43	0.99	0.90	0.79	-	-	422.00	407.00
1,2,4-trimethyl-benzene	0.8592	0.8888	47.39	51.84	1.15	1.23	0.90	0.70	1.10	1.20	424.00	413.00
1-chloro-naphthalene	1.1910	1.1717	61.67	61.67	0.75	0.99	0.53	0.55	-	3.30	357.00	387.00
Methyl-benzoate	1.1113	1.0968	64.21	62.22	0.72	0.71	0.47	0.47	3.90	3.40	368.00	380.00
Methyl-2-hydroxy-benzoate	1.1927	1.1795	71.92	71.44	0.99	0.80	0.37	0.39	6.00	-	401.00	397.00
Phenoxy-benzene	1.0730	1.0821	69.65	72.70	0.74	0.76	0.56	0.46	-	1.70	504.00	445.00
