# Supplementary material for: Aromatic Rings Commonly Used in Medicinal Chemistry: Force Fields Comparison and Interactions With Water Toward the Design of New Chemical Entities
Source: Front Pharmacol. 2018 Apr 24;9:395. doi: 10.3389/fphar.2018.00395 (PMC5928326; doi:10.3389/fphar.2018.00395)
Supplement: Supplementary file 4 [file Table_4.pdf]

Table S4. Absolute errors of calculated physical-chemical properties for each organic liquid in calibration set.

	ρ			ΔH_{vap}			Cp_{cla}			α_P			κ_T			ε			ΔG_{hyd}
Molecule Name	This work	GAFF	OPLS-AA	This work	GAFF	OPLS-AA	This work	GAFF	OPLS-AA	This work	GAFF	OPLS-AA	This work	GAFF	OPLS-AA	This work	GAFF	OPLS-AA	This work
Benzene	0.83%	-	-	4.13%	-	-	88.87%	-	-	39.12%	-	-	9.73%	-	-	52.81%	-	-	5.51%
Pyrroline	3.12%	5.68%	2.61%	6.98%	16.30%	2.24%	69.66%	151.17%	67.71%	16.60%	6.90%	20.69%	17.42%	21.54%	7.69%	48.33%	46.97%	49.49%	9.90%
Furan	7.04%	3.73%	2.89%	11.58%	11.62%	9.69%	58.95%	69.86%	63.76%	87.26%	106.85%	113.70%	-	-	-	13.35%	48.98%	48.98%	-
Fluorobenzene	2.16%	4.10%	0.23%	3.88%	3.24%	0.38%	64.69%	68.83%	57.21%	22.74%	35.59%	1.69%	6.31%	34.04%	7.45%	53.20%	38.20%	-	170.51%
1,2-fluorobenzene	2.66%	4.41%	2.67%	1.09%	5.70%	2.33%	48.62%	56.60%	71.07%	6.90%	27.50%	39.17%	-	-	-	39.80%	-	16.85%	-
1.3-fluorobenzene	4.22%	6.14%	4.72%	6.59%	7.65%	6.97%	48.01%	56.51%	63.42%	9.48%	13.33%	45.83%	-	-	-	42.65%	-	24.90%	-
1,2,3,4-fluorobenzene	4.62%	11.88%	4.79%	10.90%	3.17%	0.66%	28.49%	39.43%	42.06%	-	-	-	-	-	-	-	-	-	-
1,2,3,5-fluorobenzene	10.38%	11.56%	3.60%	3.81%	4.49%	4.80%	32.38%	45.12%	51.95%	-	-	-	-	-	-	-	-	-	-
Pyridine	3.43%	0.45%	0.26%	9.84%	3.86%	3.91%	72.50%	72.57%	71.09%	9.10%	11.76%	4.90%	29.06%	9.86%	9.86%	56.29%		48.38%	26.01%
Pyrimidine	10.69%	9.80%	7.68%	8.44%	1.33%	0.96%	67.29%	66.04%	66.04%	4.62%	28.09%	16.85%	-	-	-	-	-	-	-
Thiophene	4.16%	0.85%	2.70%	1.43%	1.13%	14.03%	48.33%	58.09%	53.25%	11.15%	27.27%	8.18%	-	-	-	45.98%	-	4.76%	70.04%
Phenol	2.43%	0.28%	0.24%	6.24%	5.61%	8.77%	48.00%	47.46%	50.42%	13.81%	43.75%	15.00%	-	-	-	41.68%	-	47.75%	16.87%
Toluene	1.00%	1.24%	1.17%	3.39%	1.58%	5.34%	54.78%	96.56%	85.75%	20.48%	51.40%	39.25%	3.16%	15.22%	4.35%	53.21%	53.59%	49.37%	25.95%
Quinoline	0.65%	0.66%	0.33%	2.43%	4.63%	5.77%	66.24%	81.00%	76.50%	3.86%	6.85%	23.29%	15.25%	11.36%	9.09%	11.98%	55.56%	55.56%	4.08%
Isoquinoline	0.43%	1.76%	0.82%	5.73%	5.38%	26.10%	72.50%	72.20%	61.56%	22.21%	23.53%	33.82%	-	-	-	70.28%	57.55%	79.25%	-
Nitro-benzene	4.19%	2.87%	2.03%	6.25%	27.87%	0.15%	58.14%	71.56%	67.04%	8.29%	9.41%	3.53%	35.37%	33.33%	5.88%	90.01%	27.61%	77.02%	106.73%
2-chloro-aniline	0.47%	2.41%	1.55%	6.56%	2.55%	0.56%	78.37%	63.04%	68.12%	-	-	-	-	-	-	70.81%	64.93%	40.30%	54.97%
Benzenethiol	5.28%	1.08%	2.04%	2.60%	9.53%	14.52%	55.65%	65.37%	59.61%	12.17%	56.82%	26.14%	4.04%	70.00%	66.00%	27.33%	32.40%	44.06%	8.91%
2-methyl-pyridine	3.49%	0.18%	0.87%	9.23%	5.64%	7.34%	45.82%	72.74%	76.51%	1.87%	3.03%	15.15%	-	-	-	51.13%	-	47.74%	17.66%
3-methyl-pyridine	1.90%	0.95%	0.13%	3.70%	1.04%	4.73%	46.55%	77.36%	83.02%	2.96%	0.00%	1.03%	-	-	-	58.53%	51.89%	39.00%	27.25%
4-methyl-pyridine	2.93%	0.00%	0.20%	6.56%	2.79%	3.55%	46.50%	76.73%	77.99%	1.07%	34.38%	1.04%	20.69%	4.29%	14.29%	53.78%	44.82%	-	20.30%
Trifluoromethyl-benzene	3.98%	0.51%	1.11%	12.92%	10.02%	2.33%	54.23%	67.37%	65.25%	7.46%	13.33%	12.50%	-	-	-	74.96%	29.50%	-	630.59%
Benzonitrile	1.08%	1.98%	0.48%	5.04%	2.65%	3.74%	56.75%	80.37%	52.15%	17.40%	26.51%	7.23%	-	-	-	48.85%	36.01%	75.01%	14.39%
Benzaldehyde	0.01%	0.64%	1.17%	9.69%	33.41%	37.55%	62.84%	69.19%	68.02%	299.00%	324.00%	208.00%	145.52%	152.17%	108.70%	54.91%	37.36%	-	36.38%
Methoxy-benzene	1.84%	0.25%	0.88%	3.24%	8.58%	5.60%	26.64%	62.03%	59.64%	5.68%	10.53%	28.42%	20.81%	4.35%	10.14%	49.52%	38.39%	43.13%	56.88%
Phenyl-methanol	0.01%	0.32%	0.04%	5.49%	4.53%	5.23%	45.42%	61.25%	74.64%	19.41%	17.39%	15.94%	-	-	-	39.30%	55.69%	39.65%	4.98%
2-methyl-phenol	2.46%	0.75%	0.65%	7.74%	11.88%	12.36%	31.31%	54.25%	58.95%	11.57%	21.52%	17.72%	-	-	-	26.39%	34.78%	34.78%	11.60%
3-methyl-phenol	2.85%	0.80%	0.98%	5.29%	7.04%	8.82%	27.54%	52.27%	62.54%	9.69%	33.33%	30.67%	18.26%	8.20%	13.11%	42.35%	50.14%	33.84%	5.40%
4-methyl-phenol	2.78%	1.92%	0.27%	2.54%	9.49%	6.55%	31.43%	51.69%	56.49%	2.67%	12.94%	4.71%	-	-	-	46.56%	65.21%	36.66%	6.82%
Ethenyl-benzene	22.74%	0.98%	1.23%	37.54%	3.41%	2.48%	40.31%	81.37%	82.47%	88.23%	17.53%	21.65%	250.40%	2.33%	13.95%	57.61%	55.28%	59.35%	135.66%
1-phenyl-ethanone	0.32%	0.19%	0.25%	1.96%	10.04%	15.51%	39.60%	91.59%	75.95%	6.58%	19.05%	5.95%	13.71%	3.57%	14.29%	58.40%	36.93%	60.44%	43.48%
Ethyl-benzene	0.71%	1.09%	0.87%	2.02%	0.26%	5.28%	39.81%	100.54%	97.30%	7.85%	32.35%	16.67%	6.73%	11.63%	3.49%	42.50%	54.73%	50.62%	120.85%
1,2-dimethyl-benzene	0.98%	1.58%	1.48%	3.35%	2.37%	6.10%	27.62%	92.55%	91.49%	10.46%	47.37%	10.53%	3.14%	20.99%	8.64%	53.60%	52.94%	41.18%	84.03%
1,2-dimethoxy-benzene	1.63%	0.65%	2.31%	27.39%	33.88%	31.31%	-	-	-	11.74%	4.30%	7.53%	-	-	-	15.99%	-	9.30%	-
2,4,6-trimethyl-pyridine	0.51%	0.25%	2.10%	4.86%	10.81%	12.71%	5.76%	77.10%	82.24%	16.48%	10.84%	7.23%	-	-	-	53.60%	48.78%	43.66%	-
(1-methylethyl)-benzene	0.52%	0.13%	1.94%	5.48%	3.90%	7.91%	40.69%	112.17%	104.63%	9.16%	45.92%	1.02%	16.57%	8.16%	19.39%	-	-	-	123.07%
1,2,4-trimethyl-benzene	1.36%	1.47%	1.93%	1.38%	0.38%	8.98%	11.66%	97.26%	92.15%	3.18%	27.78%	36.67%	15.63%	7.14%	16.67%	53.65%	53.59%	49.37%	129.28%
1-chloro-naphthalene	0.64%	0.25%	1.37%	2.78%	4.62%	4.62%	53.79%	60.71%	74.21%	11.09%	7.14%	41.43%	7.73%	8.16%	12.24%	95.05%	-	34.52%	-
Aniline	1.87%	-	-	4.80%	-	-	82.38%	-	-	46.41%	-	-	36.42%	-	-	35.93%	-	-	41.53%
Methyl-benzoate	4.00%	2.52%	1.18%	7.26%	15.55%	11.97%	37.09%	66.29%	71.71%	5.32%	18.18%	19.32%	6.07%	4.44%	4.44%	65.94%	41.27%	48.80%	39.64%
Methyl-2-hydroxy-benzoate	4.77%	0.99%	0.13%	13.75%	17.82%	17.04%	33.16%	62.01%	60.40%	5.80%	41.43%	14.29%	-	-	-	42.38%	36.64%	-	-
Phenoxy-benzene	1.77%	0.65%	1.50%	16.49%	19.22%	24.44%	68.74%	86.76%	64.89%	42.40%	13.85%	16.92%	-	-	-	47.20%	-	53.42%	-
Average absolute error	3.16%	2.20%	1.58%	7.20%	8.37%	8.83%	49.20%	72.69%	69.47%	23.88%	33.29%	25.23%	32.48%	22.67%	18.40%	49.63%	46.29%	44.75%	68.31%
Standard Deviation	3.92%	2.97%	1.54%	6.82%	8.31%	8.47%	18.49%	20.59%	13.57%	49.33%	53.04%	36.87%	58.27%	35.26%	25.68%	17.60%	10.46%	16.81%	116.19%
