# Supplementary material for: Aromatic Rings Commonly Used in Medicinal Chemistry: Force Fields Comparison and Interactions With Water Toward the Design of New Chemical Entities
Source: Front Pharmacol. 2018 Apr 24;9:395. doi: 10.3389/fphar.2018.00395 (PMC5928326; doi:10.3389/fphar.2018.00395)
Supplement: Supplementary file 5 [file Table_5.pdf]

Table S5. Deviation of calculated physical-chemical properties for each organic liquid in calibration set.

	ρ			ΔH_{vap}			Cp_{cla}			α_P			κ_T			ε			ΔG_{hyd}
Molecule Name	This work	GAFF	OPLS-AA	This work	GAFF	OPLS-AA	This work	GAFF	OPLS-AA	This work	GAFF	OPLS-AA	This work	GAFF	OPLS-AA	This work	GAFF	OPLS-AA	This work
Benzene	0.0073	-	-	-1.4061	-	-	120.60	-	-	0.48	-	-	0.09	-	-	-1.20	-	-	0.20
Pyrroline	0.0301	0.0548	0.0252	3.1529	7.3600	-1.0100	89.30	193.80	86.80	0.14	-0.06	0.18	-0.11	-0.14	-0.05	-3.83	-3.72	-3.92	1.98
Furan	0.0656	0.0347	0.0269	3.1798	3.1900	2.6600	67.67	80.20	73.20	0.64	0.78	0.83	-	-	-	-0.39	-1.44	-1.44	-
Fluorobenzene	-0.0220	-0.0418	0.0023	-1.3418	-1.1200	-0.1300	94.64	100.70	83.70	0.27	0.42	0.02	0.06	0.32	0.07	-1.99	-2.04	-	5.71
1,2-fluorobenzene	-0.0306	-0.0507	-0.0307	-0.3940	-2.0600	-0.8400	77.31	90.00	113.00	0.08	0.33	0.47	-	-	-	-5.41	-	-2.29	-
1.3-fluorobenzene	-0.0490	-0.0714	-0.0549	-2.4089	-2.8000	-2.5500	76.38	89.90	100.90	0.11	0.16	0.55	-	-	-	-2.16	-	-1.26	-
1,2,3,4-fluorobenzene	-0.0654	-0.1683	-0.0678	3.9895	-1.1600	0.2400	54.15	74.94	79.94	-	-	-	-	-	-	-	-	-	-
1,2,3,5-fluorobenzene	-0.1446	-0.1611	-0.0502	1.3497	-1.5900	1.7000	61.58	85.81	98.81	-	-	-	-	-	-	-	-	-	-
Pyridine	0.0335	0.0044	-0.0025	3.9512	1.5500	1.5700	98.31	98.40	96.40	0.09	0.12	0.05	-0.21	-0.07	-0.07	-7.31	-	-6.28	5.10
Pyrimidine	0.1087	0.0996	0.0781	4.2050	0.6600	-0.4800	89.96	88.30	88.30	0.04	0.25	0.15	-	-	-	-	-	-	-
Thiophene	0.0441	-0.0090	0.0286	-0.4944	-0.3900	4.8600	59.91	72.02	66.02	0.12	0.30	-0.09	-	-	-	-1.26	-	-0.13	4.16
Phenol	0.0256	-0.0030	0.0025	3.5169	-3.1600	4.9400	97.33	96.23	102.23	0.11	0.35	0.12	-	-	-	-4.63	-	-5.30	4.67
Toluene	0.0086	-0.0107	0.0101	-1.2864	-0.6000	2.0300	86.11	151.80	134.80	0.22	0.55	0.42	-0.03	0.14	-0.04	-1.26	-1.27	-1.17	-0.97
Quinoline	0.0071	0.0072	-0.0036	1.5583	-2.9700	-3.7000	132.49	162.00	153.00	0.03	0.05	0.17	-0.07	0.05	0.04	-1.08	-5.00	-5.00	-0.98
Isoquinoline	0.0047	-0.0192	0.0089	3.4044	3.2000	15.5100	143.15	142.55	121.55	0.15	0.16	-0.23	-	-	-	-7.45	-6.10	-8.40	-
Nitro-benzene	0.0502	0.0344	-0.0243	3.4378	15.3300	0.0800	103.02	126.80	118.80	-0.07	-0.08	-0.03	-0.18	-0.17	-0.03	-31.33	-9.61	-26.81	18.40
2-chloro-aniline	-0.0057	0.0292	0.0188	-3.7793	-1.4700	-0.3200	154.29	124.12	134.12	-	-	-	-	-	-	-9.49	-8.70	-5.40	11.29
Benzenethiol	0.0567	-0.0116	-0.0219	1.2586	-4.6200	-7.0400	96.59	113.45	103.45	0.11	0.50	0.23	0.02	0.35	0.33	1.17	-1.39	-1.89	-0.95
2-methyl-pyridine	0.0328	0.0017	0.0082	3.9614	2.4200	3.1500	72.95	115.80	121.80	0.02	-0.03	0.15	-	-	-	-5.09	-	-4.75	3.42
3-methyl-pyridine	0.0181	-0.0091	-0.0012	1.6721	0.4700	2.1400	74.01	123.00	132.00	0.03	0.00	-0.01	-	-	-	-6.81	-6.04	-4.54	5.44
4-methyl-pyridine	0.0278	0.0000	-0.0019	2.9415	1.2500	1.5900	73.94	122.00	124.00	0.01	0.33	-0.01	-0.14	-0.03	-0.10	-6.43	-5.36	-	4.19
Trifluoromethyl-benzene	0.0469	-0.0060	0.0131	4.8756	3.7800	0.8800	102.38	127.20	123.20	-0.09	0.16	0.15	-	-	-	-6.91	-2.72	-	6.60
Benzonitrile	0.0109	-0.0200	0.0048	2.6281	1.3800	1.9500	92.51	131.00	85.00	0.14	0.22	0.06	-	-	-	-12.90	-9.51	-19.81	-2.54
Benzaldehyde	0.0001	-0.0067	-0.0122	3.8368	13.2300	14.8700	108.08	119.00	117.00	0.75	0.81	0.52	0.33	0.35	0.25	-9.55	-6.50	-	6.12
Methoxy-benzene	0.0182	0.0025	-0.0087	1.4576	3.8600	2.5200	55.58	129.40	124.40	0.05	0.10	0.27	-0.14	-0.03	-0.07	-2.09	-1.62	-1.82	5.83
Phenyl-methanol	-0.0001	0.0033	-0.0004	3.6010	-2.9700	-3.4300	98.30	132.56	161.56	0.13	0.12	0.11	-	-	-	-5.14	-7.29	-5.19	1.38
2-methyl-phenol	0.0254	0.0077	0.0067	4.4056	6.7600	7.0300	73.28	126.97	137.97	0.09	0.17	0.14	-	-	-	-1.70	-2.24	-2.24	2.85
3-methyl-phenol	0.0289	0.0081	0.0099	3.2238	4.2900	5.3700	64.38	122.21	146.21	0.07	0.25	0.23	-0.11	-0.05	-0.08	-4.42	-5.23	-3.53	-1.24
4-methyl-phenol	0.0283	-0.0196	0.0028	1.6055	-6.0000	4.1400	72.11	118.59	129.59	-0.02	0.11	-0.04	-	-	-	-5.22	-7.31	-4.11	1.75
Ethenyl-benzene	-0.2049	-0.0088	0.0111	-16.4894	-1.5000	1.0900	73.57	148.50	150.50	0.86	0.17	0.21	2.15	0.02	-0.12	-1.42	-1.36	-1.46	7.04
1-phenyl-ethanone	0.0033	-0.0019	0.0026	1.0490	5.3600	8.2800	81.02	187.40	155.40	0.06	0.16	0.05	-0.08	-0.02	-0.08	-10.19	-6.44	-10.54	8.33
Ethyl-benzene	0.0061	-0.0094	0.0075	-0.8529	0.1100	2.2300	73.85	186.50	180.50	0.08	0.33	0.17	-0.06	0.10	-0.03	-1.03	-1.33	-1.23	-3.99
1,2-dimethyl-benzene	0.0086	-0.0138	0.0130	-1.4570	-1.0300	2.6500	51.93	174.00	172.00	0.10	0.45	0.10	-0.03	0.17	-0.07	-1.37	-1.35	-1.05	-3.16
1,2-dimethoxy-benzene	0.0176	-0.0070	-0.0250	13.2495	16.3900	15.1500	-	-	-	-0.11	0.04	0.07	-	-	-	-0.71	-	-0.41	-
2,4,6-trimethyl-pyridine	0.0046	-0.0023	0.0191	-2.4450	5.4400	6.4000	12.32	165.00	176.00	0.14	0.09	0.06	-	-	-	-4.19	-3.81	-3.41	-
(1-methylethyl)-benzene	-0.0045	-0.0011	0.0166	-2.4746	1.7600	3.5700	80.93	223.10	208.10	0.09	0.45	0.01	-0.16	-0.08	-0.19	-	-	-	-1.54
1,2,4-trimethyl-benzene	0.0119	-0.0128	0.0168	-0.6566	-0.1800	4.2700	25.06	209.06	198.06	0.03	0.25	0.33	-0.13	0.06	-0.14	-1.27	-1.27	-1.17	-4.65
1-chloro-naphthalene	-0.0076	0.0030	-0.0163	-1.7993	-2.9900	-2.9900	119.49	134.86	164.86	0.08	0.05	0.29	-0.04	0.04	0.06	4.79	-	-1.74	-
Aniline	0.0191	-	-	-2.6816	-	-	158.09	-	-	0.39	-	-	-0.17	-	-	-2.54	-	-	9.54
Methyl-benzoate	0.0434	0.0273	0.0128	4.0361	8.6400	6.6500	82.07	146.70	158.70	-0.05	-0.16	-0.17	-0.03	0.02	0.02	-4.38	-2.74	-3.24	6.50
Methyl-2-hydroxy-benzoate	0.0563	0.0117	-0.0015	8.3921	10.8800	10.4000	82.08	153.49	149.49	0.04	0.29	0.10	-	-	-	-4.01	-3.47	-	-
Phenoxy-benzene	0.0189	0.0069	0.0160	9.6336	11.2300	14.2800	185.50	234.13	175.13	0.28	0.09	0.11	-	-	-	-1.72	-	-1.95	-
Mean Deviation	0.0080	-0.0082	0.0010	1.5144	2.2983	3.2428	88.2009	133.8844	129.3972	0.1458	0.2238	0.1551	0.0465	0.0542	-0.0158	-4.5236	-4.2541	-4.5639	3.3488
Standard Deviation	0.0515	0.0454	0.0249	4.4567	5.4193	5.2161	33.4397	40.2247	35.3271	0.21	0.22	0.21	0.50	0.15	0.13	5.65	2.74	5.60	5.02
